# Enhanced soil health and greenhouse gas mitigation potential are linked to microbial network restructuring under long-term organic farming

**DOI:** 10.1016/j.fmre.2026.04.014

**Published:** 2026-04-23

**Authors:** Zhaoqiang Han, Ziheng Zou, Yubing Dong, Yi Leng, Shumin Guo, Zhe Xu, Longping Tu, Huacheng Yin, Yang Ruan, Shuwei Liu, Jinyang Wang, Jianwen Zou

**Affiliations:** a Key Laboratory of Agricultural Green and Low-Carbon in Southeastern China, Ministry of Agriculture and Rural Affairs, College of Resources and Environmental Sciences, Nanjing Agricultural University, Nanjing 210095, China; b Jiangsu Key Laboratory of Low Carbon Agriculture and GHGs Mitigation, College of Resources and Environmental Sciences, Nanjing Agricultural University, Nanjing 210095, China; c Jiangsu Key Lab and Engineering Center for Solid Organic Waste Utilization, Jiangsu Collaborative Innovation Center for Solid Organic Waste Resource Utilization, Nanjing Agricultural University, Nanjing 210095, China; d Huaiyin Institute of Agricultural Sciences of Xuhuai Region in Jiangsu, Jiangsu Academy of Agricultural Sciences, Huaian 223001, China

**Keywords:** Organic farming, Soil health, Greenhouse gas mitigation, Microbial community structure, Sustainable agriculture

## Abstract

Understanding how agricultural management reshapes soil microbial organization to regulate ecosystem functions and greenhouse gas (GHG) dynamics is essential for developing climate-resilient farming systems. Here, we investigated how long-term (15 years) organic farming integrating rice–duck co-culture and hairy vetch rotation affects economic profit, soil health, and GHG emissions. This assessment combined ^15^N site preference analysis of nitrous oxide (N_2_O), ^13^C-labeled and DNA-based stable isotope probing, and microbial co-occurrence network modeling. Compared with the conventional rice–wheat rotation system, the organic farming system achieved a 2.5-fold increase in net economic profit. Long-term organic management enhanced soil health by restructuring cross-domain microbial networks and altering the abundance of keystone taxa. Microbial network complexity was negatively correlated with soil health, indicating that simplified microbial interaction structures may promote soil functioning. Organic farming reduced N_2_O emissions by 65%, which was associated with a lower nitrifier-to-denitrifier gene ratio and increased *nos*Z gene abundance. Methane emissions decreased by 12%, driven by a fivefold increase in active methanotrophs. Together, these results provide trait-based evidence linking microbial community organization to soil health improvement and GHG mitigation. Our findings advance fundamental understanding of the ecological mechanisms underlying organic management effects on soil biogeochemistry and offer a theoretical basis for designing low-emission, resilient agricultural systems.

## Highlights

• Organic farming enhanced soil health and reduced N_2_O and CH_4_ emissions.
• Microbial network complexity was inversely linked with soil health under organic systems.
• Keystone microbial taxa regulated GHG emission pathways and soil processes.
• Organic system achieved 2.5-fold higher net economic returns than conventional farming.

## Graphical abstract

**Figure:**
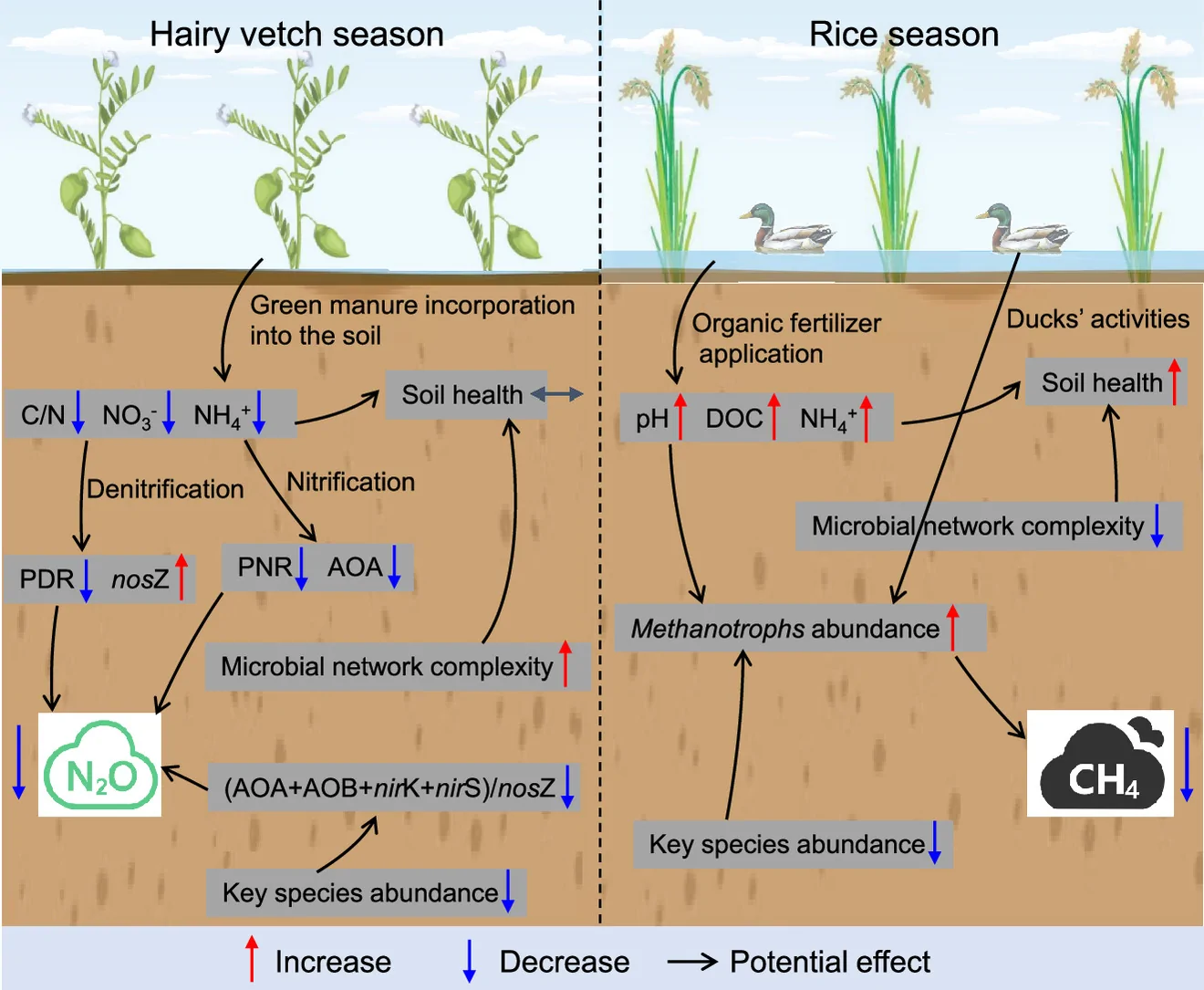

## 1 Introduction

The Green Revolution boosted agricultural productivity through the increased use of chemical inputs, including fertilizers and pesticides, together with improved irrigation, advanced soil management, and the expansion of arable land [1,2]. However, growing evidence indicates that agricultural intensification has also driven large-scale ecosystem degradation and long-term declines in sustainability. Major environmental consequences include soil degradation, increased greenhouse gas (GHG) emissions, and mounting threats to global biodiversity [3,4]. In response to these challenges, organic farming systems aim to reduce reliance on synthetic fertilizers and pesticides, lower energy inputs, and minimize soil disturbance while maintaining agricultural production [[5], [6], [7]]. Despite a surge in organic farming over the past two decades and numerous studies comparing its average performance to conventional systems [[8], [9], [10]], comprehensive assessments of its multiple ecosystem services and potential trade-offs remain limited.

A robust evaluation of organic farming contributions to environmental sustainability and food security requires the integrated assessment of key metrics, including crop yield, farmers’ economic returns, soil health, and GHG emissions [9,11]. Although existing studies suggest that organic farming can positively influence these outcomes, the long-term magnitude and consistency of these benefits remain uncertain [[12], [13], [14]]. Such variability may arise from differences in labor inputs, management intensity, and exposure to changing climatic conditions [15,16]. Moreover, few studies have identified the legacy effects of long-term organic management on soil quality and GHG emissions in comparison with conventional systems. Consequently, long-term experimental platforms are essential to capture cumulative management effects, quantify net climate impacts, assess soil ecosystem resilience, and evaluate the capacity of organic systems to sustain productivity over time.

Soil health is a holistic concept that integrates the biological, physical, and chemical properties of soil and is widely regarded as a key indicator of agroecosystem sustainability [[17], [18], [19]]. Through increased organic matter inputs and reduced soil disturbance, organic farming systems have been shown to improve soil health by increasing soil organic carbon content, nutrient retention, and microbial diversity [6,9,12,13]. However, our understanding of other potentially important drivers remains limited. In particular, how long-term organic management alters the abundance of keystone microbial taxa and the structure of microbial co-occurrence networks, and how these changes relate to soil health, is still poorly resolved. Keystone taxa, defined as species with disproportionately large ecological influence, can strongly regulate nutrient cycling, microbial interactions, and ecosystem resilience [[20], [21], [22]]. Moreover, increasing evidence suggests that the architecture of microbial co-occurrence networks, particularly cross-domain ecological networks linking bacteria and fungi, plays a vital role in regulating ecosystem multifunctionality [23,24]. Although organic management generally increases microbial diversity and biomass [25,26], its long-term impacts on microbial network complexity and keystone taxa recruitment remain unclear.

Agricultural soils are a major anthropogenic source of GHGs, accounting for 40%–50% of global CH_4_ and 25%–30% of N_2_O emissions, largely driven by the intensive N fertilization [27,28]. From a climate mitigation perspective, N_2_O and CH_4_ are of particular concern in managed agroecosystems because of their high global warming potentials and their sensitivity to management practices. Numerous studies have reported lower N_2_O emissions under organic management for most crops [14,29,30]. However, the microbial pathways responsible for these reductions remain insufficiently understood. In contrast, evidence regarding the effects of organic farming on CH_4_ emissions from rice paddies is limited and inconsistent [9]. While green manure incorporation may stimulate CH_4_ production [31,32], rice–aquaculture systems have been shown to enhance soil aeration and promote CH_4_ oxidation [33,34]. These uncertainties hinder a comprehensive evaluation of the climate mitigation potential of organic farming.

Soil microbes form the fundamental mechanistic link between management practices and soil functional outcomes [35,36]. The same microbial processes that regulate organic matter decomposition and nutrient cycling also govern the production and consumption of N_2_O and CH_4_ [37]. Therefore, a compartmentalized assessment of soil health and GHG emissions may obscure important trade-offs or synergies. For instance, does the addition of organic amendments under long-term organic farming, which typically enhances soil organic carbon and microbial biomass, also inadvertently stimulate N_2_O production by altering the microbial communities responsible for denitrification? Besides, can long-term organic farming selectively stimulate keystone taxa that simultaneously promote carbon sequestration and favor CH_4_ oxidation? To answer these questions, it is necessary to move beyond correlative studies and simultaneously quantify soil health, GHG emission potentials, and the structure and diversity of the microbial community.

To address these gaps, this study leveraged a long-term (15-year) field experiment to investigate the legacy effects of organic versus conventional management on economic performance, soil health, and GHG emissions. By integrating agronomic, biogeochemical, and microbial trait–based analyses, we evaluated how sustained organic management reshapes soil functioning and microbial organization across contrasting seasonal conditions. We tested the hypotheses that long-term organic management: (a) enhances soil health and farm profitability through improved nutrient availability and biological functioning; (b) reduces N_2_O and CH_4_ emissions by altering the composition and functional potential of key N- and CH_4_-cycling microbial communities; and (c) promotes agroecosystem sustainability by decreasing microbial network complexity and suppressing the abundance of keystone species. This study integrates economic analysis, soil health diagnostics, GHG flux measurements, isotope tracing, and microbial network modeling to holistically assess the sustainability performance of organic farming.

## 2 Materials and methods

### 2.1 Site description and sample collection

The experimental site was located at the organic rice production base of Jiangsu Oilfield Farm, Sanhe Town, Huai’an City, Jiangsu Province, China (118°47’ E, 33°10’ N). The region has a subtropical monsoon climate, with an average annual temperature of 14.9 °C and annual precipitation of 913.3 mm. Since 2008, two farming systems have been continuously implemented at this site: (a) a conventional rice-wheat rotation (C-RW) and (b) an organic rice-vetch rotation integrated with rice-duck co-culture (O-RD). Each system occupies approximately 3.5 hectares of land. Management under the C-RW system followed local traditional practices, including the use of synthetic fertilizers and pesticides. In contrast, the O-RD system adhered to organic agriculture standards, including manual weeding, prohibition of pesticides, and the exclusive use of organic fertilizers. The fertilizer types, amounts, and farming inputs can be found in Table S1. From 2008 to 2022, the average total N input under the C-RW system was 269.2 kg N ha^−1^ year^−1^ in the wheat season and 263 kg N ha^−1^ year^−1^ in the rice season, applied as urea and compound fertilizers. In the O-RD system, 251.7 kg N ha^−1^ year^−1^ was applied during the rice season using rapeseed cake and composted organic fertilizers. No N fertilizer was applied during the hairy vetch season, and all hairy vetch biomass was fully incorporated into the soil at full bloom.

Soil samples were collected on April 26 (upland season) and September 6, 2022 (rice season). To ensure robust spatial replication and avoid pseudoreplication, each management system was subdivided into five independent experimental plots (20 m × 20 m), arranged in a blocked design to account for field-scale heterogeneity. A single composite soil sample (0–20 cm depth) was collected from each plot. Each composite sample was formed by combining seven individual soil cores collected along an S-shaped transect within the plot. All samples were transported on ice and stored at 4 or −80 °C until subsequent laboratory analyses.

### 2.2 Economic benefit

The economic benefits of each farming system were evaluated based on net income, calculated as the difference between gross revenue and total input costs:

(1) $$EB=\sum \left(GB_{i}-Cost_{i}\right)$$

where *EB* denotes the economic benefit, $GB_{i}$ represents the gross benefit of agricultural product *i*, and $Cost_{i}$ refers to the total cost associated with its production. The subscript *i* includes all relevant outputs: wheat (*w*), rice (*r*), and duck (*d*). For both the conventional and organic systems, detailed records of all input items and associated expenditures were collected throughout the crop cycles. Gross revenue was calculated based on actual crop yield and local market prices at the time of harvest. The complete breakdown of farming inputs and gross benefits for each system is provided in Tables S1 and S2.

### 2.3 Soil properties determination

Soil ammonium (NH_4_^+^) and nitrate (NO_3_^–^) concentrations were extracted by using 1 M KCl and quantified colorimetrically using a UV-visible spectrophotometer (U-2900, Hitachi, Japan). Dissolved organic carbon (DOC) was extracted using deionized water (soil-to-water ratio of 1:5, w/v) and quantified with a total organic carbon analyzer (TOC-L, Shimadzu, Japan). Total N and C contents were analyzed using a EuroEA 3000 elemental analyzer (EuroVector, Milano, Italy). Soil pH was measured using a pH detector (PHS-3C mv/pH detector, Shanghai, China) in a 1:2.5 soil-to-water suspension.

Eight key soil enzymes involved in nutrient cycling were quantified: cellobiohydrolase (CBH), acetylglucosaminidase (NAG), β-1,4-glucosidase (BG), acid phosphatase (AP), leucine aminopeptidase (LAP), phenol oxidase (PPO), and peroxidase (PER). CBH, NAG, BG, and AP activities were measured fluorometrically using 4-methylumbelliferone-labeled substrates in black 96-well microplates using a microplate reader (Varioskan LUX, Thermo Fisher, USA). LAP activity was determined using 7-amino-4-methylcoumarin as the substrate [38]. PPO and PER were assayed colorimetrically in clear 96-well plates using L-3,4-dihydroxyphenylalanine (L-DOPA) as the substrate. Urease activity was measured by the indophenol blue colorimetric method after incubation with 10% urea solution, following the protocol of Guan et al. [39].

### 2.4 Soil health assessment

The soil health status of each cropping system was quantified using the Cornell Soil Health Assessment (CSHA) framework [40]. A total of fourteen soil indicators representing physical, chemical, and biological properties were included in the assessment. These indicators comprised: soil pH, total N, total C, C/N ratio, NH_4_^+^, NO_3_^-^, DOC, BG, NAG, AP, LAP, PPO, PER, and urease. The rationale for their selection is presented in Table S3. Each indicator was normalized and converted into a CSHA score (A) based on standard reference ranges [41]. The weighting value of individual soil indicators was based on Principal Component Analysis (PCA) of all soil properties (Fig. S2c, d). These individual scores were then integrated into a composite soil health index using the following weighted average formula:

(2) $$\text{Soil}\;\text{health}\;\text{score}=\;\frac{A_{1}\times w_{1}+A_{2}\times w_{2}+\cdots +A_{n}\times w_{n}}{w_{1}+w_{2}+\cdots +w_{n}}$$

where *A*_i_ is the normalized score for the *i*th indicator, and *w*_i_ is its corresponding weighting factor. The weights reflect the relative importance of each indicator in maintaining soil functionality and ecosystem service delivery. Detailed normalization procedures, scoring scales, and weighting schemes are provided in the Supplementary Methods.

### 2.5 GHG emissions potential

To evaluate the potential GHG emissions under different cropping systems, soil incubation was conducted separately for the upland and rice seasons using fresh soil samples. For each sampling event, 50 g of fresh soil (dry-weight equivalent) was placed into 250 mL Erlenmeyer flasks. For upland soils, 10 mg of urea was applied to each flask, and deionized water was added to adjust the soil moisture content to 60% of its water-holding capacity to observe N_2_O and CO_2_ fluxes. For rice-season soils, the same amount of urea was applied, and deionized water was added to create a 2 cm flooding depth to simulate paddy field conditions to measure CH_4_ and CO_2_ fluxes. All flasks were incubated aerobically in the dark at 25 °C. Deionized water was replenished every 2 days to compensate for evaporation and to ensure constant moisture or flooding depth throughout the incubation period. Headspace gas samples were collected at 2-day intervals for the first 21 days and less frequently thereafter, continuing up to day 42 (the incubation was concluded after 42 days when GHG fluxes from all treatments had stabilized at levels near the detection limit). CO_2_, N_2_O, and CH_4_ concentrations were analyzed using gas chromatography (Agilent 7890A, USA), and emission fluxes were calculated based on the linear change in gas concentration over time within each flask. Cumulative emissions were calculated by integrating flux data over the full incubation period. The calculation formulas for emission fluxes are presented in Supplementary Methods. Due to the results showing that the cumulative CO_2_ emissions during the whole incubation experiment had no significant difference between C-RW and O-RD treatments (*P* < 0.05; Fig. S3), we focused the incubated GHG flux analysis specifically on N_2_O and CH_4_.

### 2.6 Microbial-derived production and emission potentials of N_2_O and CH_4_

Soils collected during the upland season were used to determine potential nitrification (PNR) and denitrification rates (PDR). For PNR, three replicates of 3 g fresh soil (dry-weight equivalent) were placed into three 120 mL serum bottles, each amended with 20 mL of substrate solution containing 0.2 M K_2_HPO_4_, 0.2 M KH_2_PO_4_, and 0.02 M NH_4_SO_4_ [42]. The bottles were sealed and incubated under aerobic conditions at 25 °C. For PDR, 3 g of fresh soil was placed into 25 mL serum bottles containing denitrification buffer. Bottles were vortexed to form a uniform slurry and sealed with rubber stoppers. To block N_2_O reduction to N_2_, the headspace was evacuated to <0.1 atm using a vacuum pump and replaced with 25 mL of C_2_H_2_ (10% *v*/*v*) [43]. Incubations were conducted at 25 °C under anaerobic conditions, and headspace N_2_O concentrations were measured over time. The detailed method for determining PNR and PDR can be found in Supplementary Methods.

Soils sampled during the rice season were used to determine CH_4_ production and oxidation potentials. For measuring soil CH_4_ production potential, 2.5 g dry weight of fresh soil was weighed and spread flat on the bottom of a 25 mL serum bottle, 5 mL of deionized water was added to provide a 1 cm water layer on top of the soil, and the bottle was sealed with a rubber stopper. After pumping the air pressure in the bottle to below 0.1 atm with a vacuum pump, 25 mL of high-purity N_2_ was added. The treated samples were placed in an incubator at 25 °C, and 2 mL of gas was collected at 2, 48, and 72 h. The soil CH_4_ oxidation potential was characterized by the amount of CH_4_ oxidized per unit time of soil under methane-saturated conditions. The experimental detail was as follows: 10 g of fresh soil was added to a 150 mL serum bottle, which was sealed with a rubber stopper after adding 10 mL of pure water. The bottle was filled with 1.5 mL of CH_4_ (1%, 10,000 ppmv), placed on a shaker, and incubated for 2 h at a constant temperature of 25 °C, protected from light, and then 2 mL of gas was taken as the initial concentration of CH_4_. Then 2 mL of gas was collected after 12, 24, and 48 h, respectively. The amount of CH_4_ produced per unit of time was calculated by linear fitting as the CH_4_ production potential, while the amount of CH_4_ oxidized per unit of time was calculated by linear fitting as methane oxidation potential (μg kg^−1^ h^−1^) [44]. The detailed measurements for CH_4_ production and oxidation potentials are presented in Supplementary Methods.

### 2.7 Isotopic analysis and DNA-SIP stable-isotope probing

On day 20 of the soil incubation described in Section 2.3, when N_2_O emission rates peaked, 20 mL of headspace gas samples were collected from each flask to determine the isotopic composition of N_2_O. Measurements of δ^15^N, δ^18^O, and N_2_O isotopomers (δ^15^N^α^ and δ^15^N^β^) were conducted using a gas chromatograph-isotope ratio mass spectrometer (GC-IRMS, Thermo Fisher Scientific Co., Ltd., Beijing, China). The relative contributions of nitrification (*Ni*), nitrifier-denitrification (*nD*), bacterial denitrification (*bD*), and fungal denitrification (*fD*) to N_2_O production were estimated based on dual-isotope signatures and SP values. Additionally, the extent of N_2_O reduction to N_2_ was inferred using two-pool models: reduction-mixing and mixing-reduction [45,46]. Detailed procedures for isotopocule modeling, SP correction, and process attribution are described in the Supplementary Methods.

DNA-based stable isotope probing (DNA-SIP) was employed to trace active methanogenic and methanotrophic communities. For measuring active methanogenic, 10 g of fresh soil were weighed in duplicate into 120 mL serum bottles and amended with 50 mL of ^13^C-glucose solution (140 mg C L^−1^, 99 atom%, Sigma-Aldrich) or ^12^C-glucose for controls. Bottles were sealed with rubber stoppers, flushed with N_2_ for 15 min to create anaerobic conditions, and incubated in the dark at 25 °C for 2 weeks. For measuring active methanotrophs, fresh soils (10 g) were placed into 120 mL serum bottles with moisture adjusted to 60% water-holding capacity using deionized water. Bottles were sealed and flushed with ambient air, then injected with either 2.5 mL of ^13^CH_4_ (99 atom%) or ^12^CH_4_ as a control. The sealed bottles were incubated aerobically in the dark at 25 °C for 2 weeks. In both incubation experiments, 10 mL gas was collected from each bottle at 1, 2, 3, 5, 7, 9, 11, 13, and 15 days to determine the CH_4_ concentrations using chromatography according to the method in 2.3. After each sampling event, an equal amount of N_2_ and standard air was injected into corresponding bottles to maintain the appropriate conditions for CH_4_ production and oxidation, respectively [44]. The CH_4_ concentrations during the respective DNA-SIP incubation are shown in Fig. S1.

After 2 weeks of ^13^C-labeled substrate incubation, soils were sampled to identify actively metabolizing microbial populations. For each treatment, 0.25 g of soil from both ^13^C- and ^12^C-incubated bottles was used for DNA extraction using the FastDNA™ Spin Kit for Soil (MP Biomedicals, USA). Extracted DNA was dissolved in 100 μL of sterile water, and concentrations were measured with a NanoDrop ND-1000 microvolume spectrophotometer. To separate ^13^C-labeled DNA, approximately 2 μg of total DNA was mixed with cesium chloride (CsCl) solution to achieve an initial buoyant density of 1.725 g mL^−1^. The DNA-CsCl solution was loaded into 5.1 mL ultracentrifuge tubes and centrifuged at 45,000 rpm (190,000 × g) at 20 °C for 44 h using a Beckman Coulter Optima ultracentrifuge (Optima XPN, USA). After centrifugation, the solution was fractionated into 15 equal-volume layers from bottom to top. DNA in each layer was precipitated using PEG-6000, washed with 70% ethanol, and resuspended in 30 μL of sterile water. Labeled (^13^C-enriched) DNA was subsequently identified based on buoyant density shifts and used for downstream quantification and sequencing of active methanogen and methanotroph communities [47,48].

### 2.8 Real-time fluorescence quantitative PCR and high-throughput sequencing

Quantitative real-time PCR (qPCR) reactions were carried out using SYBR® Premix Ex Tag™ kit (Takara, Japan) in 96-well plates on a Step One Plus™ Real-Time PCR System (Applied Biosystems, CA, USA). DNA templates included both total genomic DNA from bulk soil and ^13^C-labeled DNA obtained from the DNA-SIP experiments. For soils collected during the upland season, the following genes were quantified: ammonia-oxidizing archaea (AOA), ammonia-oxidizing bacteria (AOB), *nir*S, bacterial *nir*K, and *nos*Z. For soils collected during the rice season, functional genes related to CH_4_ cycling were assessed: *mcr*A and *pmo*A. Gene-specific primers and thermal cycling conditions followed established protocols and are detailed in Table S4. Each reaction was performed in triplicate, and gene copy numbers were normalized to dry soil weight.

To characterize microbial community composition, amplicon sequencing was performed on the 16S rRNA gene for bacteria and archaea, and the internal transcribed spacer (ITS) region for fungi. Additionally, targeted sequencing was performed on the *mcr*A and *pmo*A genes amplified from the labeled DNA fractions obtained in Section 2.7. Raw sequencing data were processed using the QIIME2 pipeline. Adapter and primer sequences were removed using the Cutadapt plugin. Quality filtering and amplicon sequence variant (ASV) inference were conducted using the DADA2 plugin. Taxonomic classification of representative sequences was performed using a Naive Bayes classifier trained on the SILVA (for 16S) and UNITE (for ITS) reference databases, with a confidence threshold set at 0.8. All Illumina raw sequences of bacterial and fungal communities were submitted to the NCBI Sequence Read Archive (SRA) under BioProject accession number PRJNA1276277.

### 2.9 Microbial network construction

To explore microbial interaction patterns across domains (bacteria and fungi), co-occurrence networks were constructed using the ‘*ggClusterNet*’ package in R (version 4.5.0) following the method described by Wen et al. [49]. Only operational taxonomic units (OTUs) with relative abundances greater than 0.01% were retained for network analysis to reduce noise and improve biological relevance. Pairwise Spearman correlation coefficients were calculated between OTUs. OTU pairs with correlation coefficients > 0.6 and *P*-values < 0.05 were considered significant and included as edges in the network. The random matrix theory (RMT) was used for constructing co-occurrence networks. RMT was achieved to identify 0.85 and 0.80 as the thresholds for bacterial and fungal networks in the rice season, and 0.86 and 0.74 as the thresholds for bacterial and fungal networks in the upland season, respectively. All networks were visualized using Gephi (version 0.9.2).

Key topological properties calculated for each network included: the total number of edges and nodes, proportions of positive and negative correlations, connectance, average degree, average path length, and centralization metrics (i.e., degree, betweenness, and closeness). To quantify network complexity, a composite index was calculated by averaging the standardized Z-scores of these topological features, following the method of Long et al. [50]. The ecological roles of individual nodes were further categorized based on within-module connectivity (Zi) and among-module connectivity (Pi). Specifically, nodes with Zi > 2.5 were classified as module hubs; nodes with Zi > 2.5 and Pi > 0.62 as network hubs; nodes with Zi < 2.5 and Pi > 0.62 as connectors; and nodes with Zi < 2.5 and Pi < 0.62 as peripheral nodes [51]. Keystone taxa were explicitly defined as all OTUs classified as module hubs, network hubs, and connectors. This combined group is theorized to play a disproportionately large role in maintaining network structure and stability due to their high connectivity [51].

### 2.10 Statistical analyses

All statistical analyses were conducted in R (v4.5.0). Differences between treatment means were assessed using two-group comparisons with independent two-sample t-tests using the ‘*dplyr’* package. Before t-tests, the assumptions of normality (Shapiro–Wilk test) and homogeneity of variances (Levene’s test) were checked. For data that violated these assumptions, the non-parametric Mann–Whitney U test (Wilcoxon rank-sum test) was used instead. The results of these tests are reported with the corresponding t-/U-statistic, degrees of freedom (where applicable), and *P*-values. Partial least squares path modeling (PLS-PM) was used to explore causal relationships among farming practices, microbial traits, soil health, and GHG emissions. PLS-PM was conducted using the ‘*plspm*’ R package, and standardized total effects were reported. The performance and reliability of the PLS-PM were evaluated using the Goodness-of-Fit (GOF) index, which was calculated as the geometric mean of the average communality (outer model) and the average *R*² (inner model). A higher GOF indicates better overall model performance in explaining the data. The PCA analyses were conducted using the ‘*FactoMineR*’ R package.

## 3 Results

### 3.1 Effects of organic farming on soil health and economic benefits

Soil health was evaluated using the CSHA framework, incorporating 14 physicochemical and biological indicators (Table S5). Under the O-RD system, soil pH increased significantly in both seasons, while NO_3_^–^ accumulation declined in the upland season. Additionally, DOC content increased markedly during the rice season. PCA captured 70.1–76.1% of the total variance across sampling periods, with a clear separation between the two systems (Fig. S2). Overall, O-RD management increased the composite soil health score by 51% during the rice season (*P* < 0.001; Fig. 1). However, no significant differences in soil health were observed during the upland season. Organic farming significantly improved both economic returns and soil health compared to the C-RW treatment. Specifically, the O-RD system achieved a 2.5-fold increase in net economic profit relative to the C-RW system (*P* < 0.001; Fig. S4), primarily driven by higher market prices for organic rice products (Table S2).

**Fig. 1:**
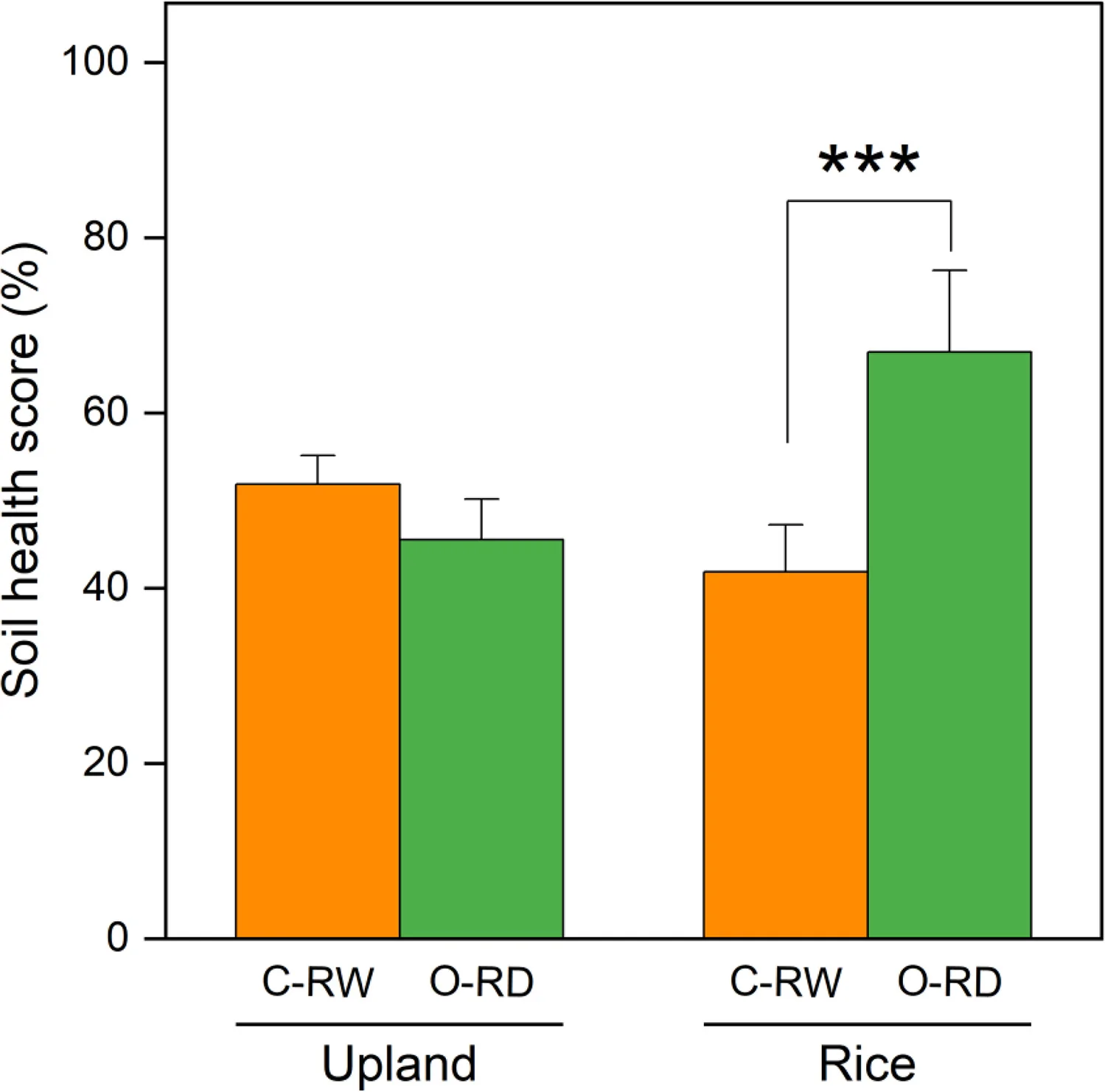
**Soil health assessment for different cropping patterns.** Treatment abbreviations: C-RW conventional rice-wheat rotation, O-RD organic rice-vetch rotation combined with rice-duck co-culture. ^⁎⁎⁎^*P* < 0.001.

### 3.2 Organic farming reduced the N_2_O emission potential

The organic regime significantly decreased N_2_O emissions during the upland season by regulating microbial N transformation processes. Throughout the incubation period, soils under the C-RW system consistently exhibited higher N_2_O fluxes than those under the O-RD system (Fig. 2a). Cumulative N_2_O emissions were reduced by 65% in the O-RD compared to C-RW (*P* < 0.001; Fig. 2b). Soil PNR in C-RW soils was nearly ten times greater than in O-RD, while the PDR was elevated by 68% under organic management (Fig. 2c, d). This dual response-suppressed nitrification and enhanced denitrification (N_2_O→N_2_)-indicated a shift in dominant N_2_O pathways under O-RD.

**Fig. 2:**
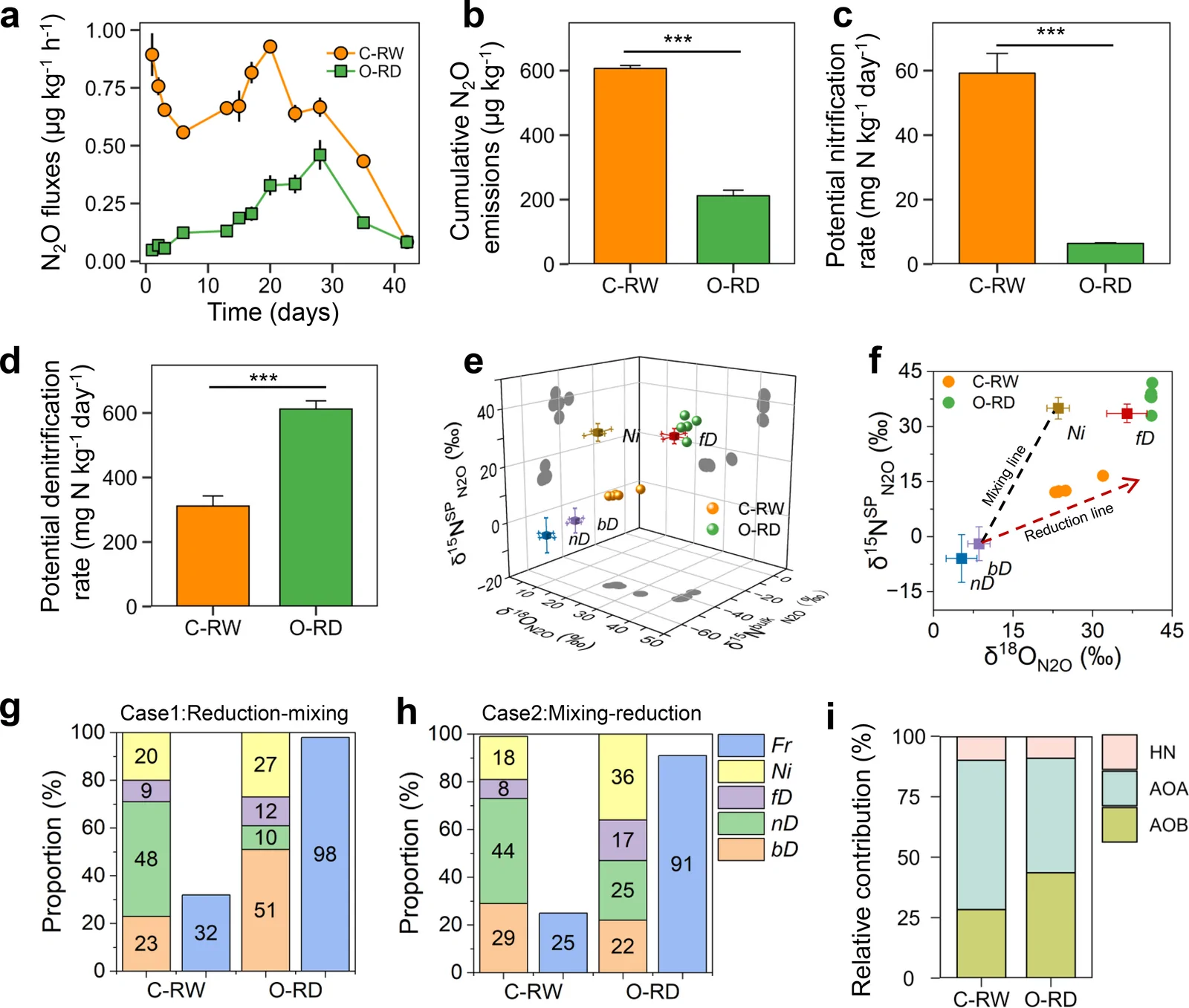
**N_2_O emissions potential and related key drivers for different cropping patterns.** (a) N_2_O fluxes for the two cropping patterns during the incubation period. (b) cumulative N_2_O emissions for the two cropping patterns during the incubation period. (c-d) potential nitrification (PNR) and denitrification (PDR) rates for different cropping patterns. (e) The 3D map for δ^15^N^SP^ (z- axis), δ^18^O (x- axis), and δ^15^N^bulk^ (y- axis). N_2_O mixing endmembers form four different colored cubes, representing the expected ranges for bacterial nitrification (*Ni*), fungal denitrification (*fD*), bacterial denitrification (*bD*), and nitrifier denitrification (*nD*), as shown in Table S6. The gray solid dots are the projections of all the N_2_O isotope points. (f) SP versus corrected δ^18^O-N_2_O dual isotopocule mapping. Boxes represent the expected ranges for *Ni, fD, bD*, and *nD*. The dashed black line shows the alternative bD-Ni mixing line. The dashed red line shows the minimum reduction line for the case of minimal delta values of the bD endmember. The detailed calculation strategy mapping approach can be found in the Supplementary Methods. (g-h) Different pathways fraction of N_2_O production (*Ni, fD, bD*, and *nD*) and N_2_O reduction fraction in two modeling cases. The calculations are performed according to two possible cases of N_2_O mixing and reduction. The detailed calculation strategy can be found in the Supplementary Methods. (i) The contribution of AOA, AOB, and heterotrophic nitrification (HN) for N_2_O emission during the nitrification process. In b–c, the whiskers in each column indicate the standard deviation of five replications.

Three-dimensional and dual-isotope mapping (SP vs. δ^18^O) revealed that samples clustered within reduction–mixing trajectories (Fig. 2e, f), enabling quantitative attribution of N_2_O sources. Process modeling using both the reduction–mixing and mixing–reduction frameworks demonstrated a markedly reduced contribution of nitrifier denitrification (*nD*) under O-RD, and an increased proportion of N_2_O reduction to N_2_. Specifically, the estimated N_2_O reduction fraction in O-RD soils exceeded 90% in both models, significantly higher than in C-RW (Fig. 2g, h). Besides, the O-RD system reduced the contribution of AOA to nitrification relative to C-RW (Fig. 2i).

The ratio of (AOA + AOB + *nir*K + *nir*S) to *nos*Z gene abundances was calculated as an index of the genetic potential for net N_2_O production. A higher ratio indicates a microbial community where the genetic capacity for N_2_O production via nitrification and denitrification is proportionally greater than the capacity for its reduction to N_2_. Although O-RD reduced AOA abundance compared to C-RW, it significantly increased the abundance of AOB and *nos*Z genes (*P* < 0.05; Fig. S5). Although reductions in *nir*K and *nir*S (N_2_O production genes) were modest, the combined ratio of N_2_O-producing to N_2_O-reducing genes (i.e., (AOA + AOB + *nir*K + *nir*S)/*nos*Z) was significantly lower in O-RD (*P* < 0.001; Fig. S5).

### 3.3 Organic farming mitigates soil CH_4_ emission potential

A 42-day laboratory incubation showed that the organic regime reduced CH_4_ emissions in paddy soils, despite comparable temporal emission patterns between systems (Fig. 3). The cumulative CH₄ emission under the O-RD system was reduced by 12% compared to C-RW (*P* < 0.05; Fig. 3b). No significant differences were observed in CH₄ production/oxidation potentials between the two systems (Fig. 3c, d). The success of the DNA-SIP incubations and the specific isotopic labeling of microbial taxa were confirmed by quantitative PCR and sequencing of the gradient fractions (Tables S7, S8). ^13^C-DNA-SIP analysis showed that the abundance of active methanogens remained unchanged between treatments, but the relative abundance of active methanotrophs increased by more than fivefold under O-RD (Fig. 3e,f). Gene abundance analysis further showed that although the abundances of *mcr*A and *pmo*A did not differ significantly between treatments (Fig. S6), the ratio of *mcr*A to *pmo*A, a proxy for the relative dominance of CH_4_ production versus oxidation, tended to decrease in O-RD soils, albeit not significantly (Fig. S6).

**Fig. 3:**
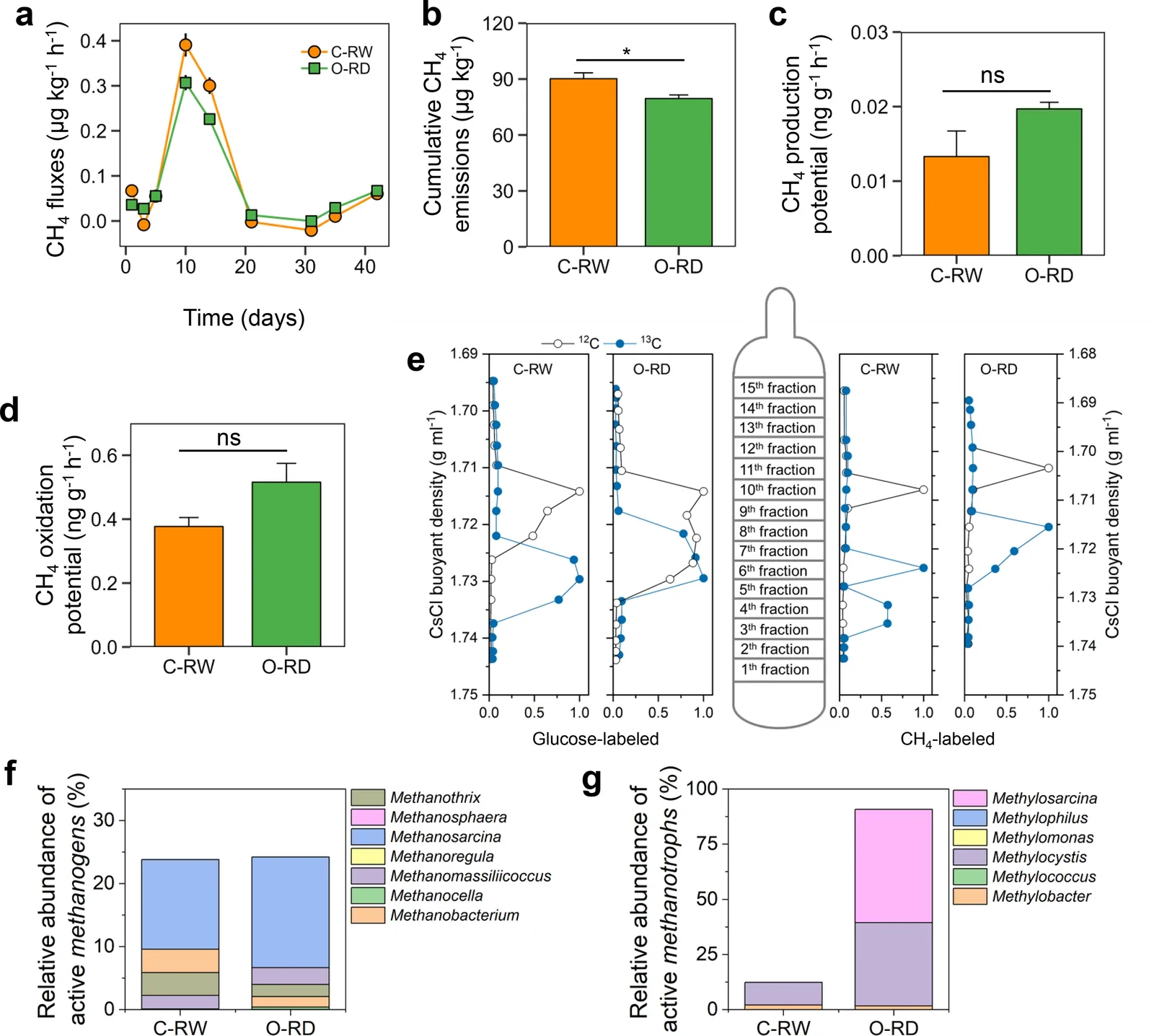
**CH_4_ emissions potential and related key drivers for different cropping patterns.** (a) CH_4_ fluxes for the two cropping patterns during the incubation period. (b) cumulative CH_4_ emissions for the two cropping patterns during the incubation period. (c-d) CH_4_ production and oxidation potential. (e) Distribution of ^13^C-labelled methanogens (left) and methanotrophs (right). The data have been normalized to represent the ratio of gene abundance in each DNA gradient to the maximum number for each treatment. (f-g) The relative abundance of active methanogens and methanotrophs in different cropping patterns. In b-c, the whiskers in each column indicate the standard deviation of five replications.

### 3.4 The changes in microbial diversity, community structure, and key species abundance under organic farming

Compared to C-RW, the O-RD system did not significantly affect bacterial diversity but led to a marked increase in fungal diversity by 15% in the upland season and 47% in the rice season (Fig. S7c, g). Principal coordinate analysis (PCoA) confirmed significant shifts in microbial community composition across both systems and seasons (*P* = 0.001). During the upland season, the first two PCoA axes explained 85.7% of the variation in bacterial communities and 94.3% in fungal communities (Fig. S7b, d). Similarly, during the rice season, 94.3% and 90.1% of community variation in bacteria and fungi, respectively, was explained (Fig. S7f, h). At the phylum level, dominant bacterial groups included *Actinobacteriota, Chloroflexi, Firmicutes, Proteobacteria, Acidobacteriota, Myxococcota, Verrucomicrobiota, Planctomycetota*, and *Desulfobacterota,* together accounting for over 90% of total sequences. Fungal communities were dominated by *Ascomycota, Zygomycota, Basidiomycota*, and Fungi_unclassified, which comprised over 99% of total fungal sequences (Fig. S7i, j).

Microbial cross-domain co-occurrence networks (bacteria–fungi) revealed that in the upland season, O-RD significantly increased overall network complexity compared to C-RW (*P* < 0.05; Fig. 4a, b, e). In contrast, in the rice season under organic management, no significant differences in network complexity were observed between systems (Fig. 4c, d, g). Across both seasons, soil health scores were negatively correlated with microbial network complexity (Fig. 4f, h), suggesting that more simplified (less entangled) networks may reflect greater soil health under organic management. Further analysis of keystone taxa showed that 31 and 29 OTUs formed the cross-domain ecological networks in the upland and rice seasons, respectively (Fig. S8a, d; Tables S9, S10). In the upland season, O-RD increased the relative abundance of both bacterial and fungal keystone taxa (e.g., *Actinobacteriota, Chloroflexi, Firmicutes, Ascomycota, Basidiomycota, Zygomycota*; Fig. S8b, c). In contrast, during the rice season, O-RD increased key fungal taxa but reduced the abundance of key bacterial groups (Fig. S8e, f).

**Fig. 4:**
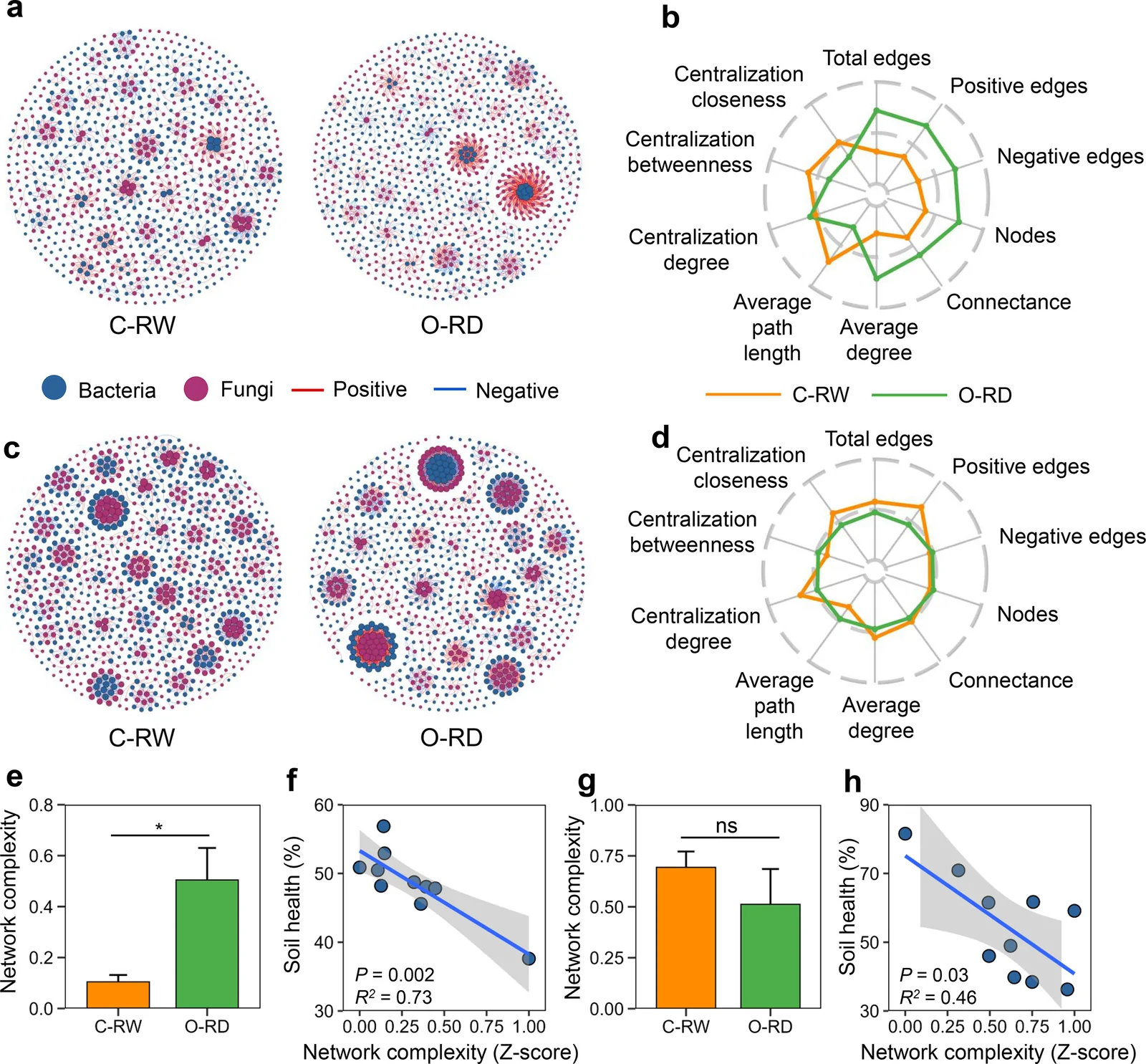
**Microbial co-occurrence networks and related topological features.** (a) and (c) the inter-domain ecological network of bacteria and fungi in the upland and rice seasons, respectively. (b) and (d) the difference of related topological features between the two systems in the upland and rice seasons, respectively. (e) and (g) the network complexity in different cropping systems. (f) and (h) the relationship between microbial network complexity and soil health in the upland and rice seasons, respectively. The results of “*”, “**”, and “***” represent *P* < 0.05, *P* < 0.01, and *P* < 0.001, respectively, and “ns” represents there is no significant difference between the two systems.

### 3.5 Keystone species-mediated organic farming effects on GHG emissions

Pearson correlation analyses revealed that the abundance of keystone species in microbial co-occurrence networks significantly influenced soil health and GHG emissions (Fig. 5a, c). In the upland season, PLS-PM analyses suggested that organic farming had a significant and negative effect on the abundance of key species (*P* < 0.01; Fig. 5b), which in turn indirectly led to a decline of soil N_2_O emissions by decreasing the ratio of (AOA + AOB + *nir*K + *nir*S)/*nos*Z. Besides, soil health could directly increase the ratio of (AOA + AOB + *nir*K + *nir*S)/*nos*Z in the upland season. Organic farming had the highest total negative effect, while the ratio of (AOA + AOB + *nir*K + *nir*S)/*nos*Z had the highest total positive effect on N_2_O emissions (Fig. 5e). In the rice season, there was a negative correlation between the abundance of key species and methanotrophs (Fig. 5d). Organic farming could indirectly lower soil CH_4_ emissions by positively influencing the abundance of methanotrophs through the negative effect on the abundance of key species (Fig. 5d). Organic farming could also directly induce the improvement of soil health in the rice season. Organic farming and the abundance of methanotrophs and key species exerted overall negative effects on CH_4_ emissions in the rice season (Fig. 5f). However, whether in the upland or rice season, our PLS-PM analysis results did not find a direct or indirect effect of key species abundance on soil health.

**Fig. 5:**
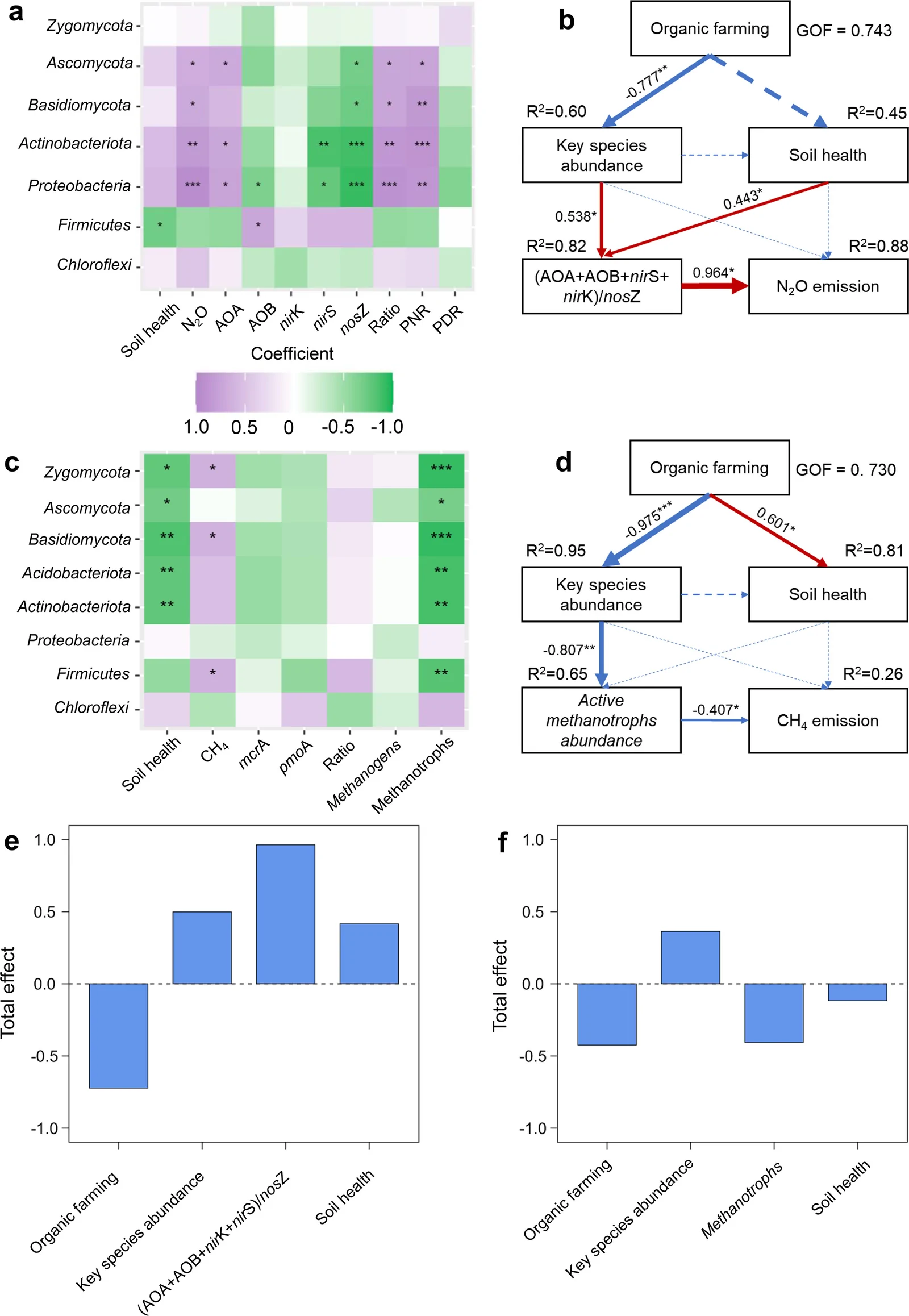
**The relationships between the abundance of key species, soil health, and GHG emissions.** (a) Pearson correlation between key species abundance and soil health, N_2_O emissions, N-cycling genes abundance, PNR, and PDR in the upland season. (b) and (d) Partial Least Squares Path Modeling (PLS-PM) describing the effects of organic farming on soil health, N_2_O, and CH_4_ emissions. The red and blue lines represent positive and negative effects, respectively. Numbers adjacent to arrows are indicative of the strength of the causal relationship. Significance levels of each predictor are *P < 0.05, **P < 0.01, and ***P < 0.001 (c) Pearson correlation between key species abundance and soil health, CH_4_ emissions, the abundances of *mcr*A, *pmo*A, methanogens, and methanotrophs. (e) and (f) the standardized total effects (direct plus indirect effects) of related factors on N_2_O and CH_4_ emissions derived from PLS-PM used, respectively. In b, the abundance of key species represents the total abundance of *Ascomycota, Basidiomycota, Actinobacteriota*, and *Proteobacteria*. In d, the abundance of key species represents the total abundance of *Zygomycota, Ascomycota, Basidiomycota, Acidobacteriota*, and *Actinobacteriota. R^2^* values denote the amount of variance explained by the model for the response variables.

## 4 Discussion

### 4.1 Organic farming improves soil health and profitability

Soil health is a foundational component of agricultural productivity, ecosystem resilience, and climate regulation [18,52,53]. Using a comprehensive set of physicochemical and biological indicators, we show that 15 years of organic management significantly enhanced soil health, particularly in the rice season. Concurrently, the organic system yielded more than a threefold increase in annual net income, primarily driven by the premium market value of organically certified rice (Fig. S4). When actual price premiums are considered, organic farming has been shown to achieve higher profitability and benefit-cost ratios than conventional agriculture [6]. In the upland season, the C-RW system exhibited a slightly but insignificantly higher soil health index than O-RD (Fig. 1). This marginal advantage likely stems from elevated soil mineral N and total N content under the C-RW system (Table S5), which can temporarily enhance soil nutrient availability [54]. However, this apparent short-term benefit was accompanied by clear signs of soil degradation. Long-term synthetic fertilizer application led to soil acidification under C-RW, with pH declining to 4.84 [55]. Additionally, lower C/N ratio and higher PER activity were observed (Table S5), suggesting accelerated decomposition of recalcitrant organic matter and a potential decline in soil organic carbon stocks [56]. In contrast, the O-RD system maintained higher total C and N contents in the rice season, and exhibited more neutral soil pH conditions, together with increased NH_4_^+^ availability. These conditions are favorable for flooded rice systems, where NH_4_^+^ is the dominant form of nitrogen uptake. Increased oxidative enzyme activities further suggest enhanced lignin degradation and organic matter turnover under organic management [56,57]. Collectively, these patterns provide direct support for our first hypothesis, confirming that long-term organic management can improve soil health while simultaneously increasing farm profitability.

A key finding of our study was that significant improvements in soil health indicators under organic management were consistently more pronounced during the rice season compared to the upland season. We attribute this seasonal contrast to the interaction between management practices and environmental conditions. Flooded, anaerobic conditions during the rice season likely slow the decomposition of applied organic amendments, promoting their accumulation as more stable soil organic matter and fostering microbial communities adapted to anaerobic metabolism and slower carbon turnover [58,59]. By contrast, the return to oxic conditions and higher temperatures during the upland season can accelerate the mineralization of both native and added organic matter, thereby reducing detectable differences between management systems [60]. This suggests that the upland season may represent a period of heightened carbon vulnerability. Management strategies targeted to this phase, such as cover cropping or reduced tillage, may therefore be necessary to fully realize the soil health benefits of organic farming in rice-upland rotation systems.

### 4.2 Mechanisms of GHG mitigation under organic farming

Our results demonstrate that long-term organic farming can substantially reduce soil N_2_O and CH_4_ emission potentials by altering microbial N and C transformation pathways. Because the majority of soil N_2_O emissions in rice-upland rotation systems occur during the upland season [61,62], we focused our analysis on this critical period. Cumulative N_2_O emissions were 65% lower under the O-RD system than C-RW (Fig. 2b). This reduction was linked to two complementary microbial mechanisms: suppression of ammonia oxidation and enhancement of complete denitrification. Isotopocule analysis revealed that O-RD soils had lower contributions from nitrifier-denitrification pathways and higher proportions of N_2_O reduction to N_2_. Estimated reduction fractions exceeding 90% in both reduction-mixing and mixing-reduction models (Fig. 2g, h). This was consistent with the observed decline in the abundance of AOA, and the functional gene ratio (AOA + AOB + *nir*K + *nir*S)/*nos*Z under O-RD management (Fig. S5f). These findings support previous studies suggesting that elevated *nos*Z abundance promotes complete denitrification and limits net N_2_O accumulation [62]. Furthermore, organic soils had higher C/N ratios and lower NO_3_^–^ concentrations than conventional soils (Table S5), conditions that favor C-limited denitrification while restricting N_2_O-producing pathways. Increased PDR under O-RD further indicates an enhanced microbial capacity for N_2_O reduction, consistent with elevated *nos*Z abundance (Figs. 2d and S5e). Collectively, these results support our second hypothesis that long-term organic farming reduces N_2_O emissions by directing microbial functional pathways toward greater reduction efficiency.

The O-RD system also reduced cumulative CH_4_ emissions by 12% relative to C-RW (Fig. 3b), despite similar CH_4_ production and oxidation potentials between the two systems (Fig. 3c, d). This discrepancy suggests that differences in net CH_4_ emissions were not driven by gross microbial capacity but instead reflected shifts in active functional populations. DNA-SIP analysis showed that the abundance of active methanotrophs increased fivefold under organic management (Fig. 3g), while methanogen abundance remained unchanged (Fig. 3f). This selective enrichment was accompanied by a moderate decline in the *mcr*A/*pmo*A ratio (Fig. S6c), indicating a shift in the microbial balance toward enhanced CH_4_ oxidation. We attribute the enrichment of methanotrophs primarily to the altered aeration conditions associated with rice-duck co-culture. Although direct measurements of soil oxygen were not conducted, duck paddling and foraging activities are known to disrupt the soil-water interface, promote oxygen diffusion into surface sediments, and create transient aerobic microsites that stimulate CH_4_ oxidation [63,64]. Additionally, higher DOC and NH_4_^+^ availability under O-RD may have further favored methanotroph proliferation while limiting acetate-dependent methanogenesis [65,66]. Together, these findings indicate that organic management suppresses both N_2_O and CH_4_ emissions by jointly regulating substrate availability and microbial guild composition, thereby confirming our second hypothesis and elucidating the biogeochemical mechanisms underlying organic farming-driven GHG mitigation.

### 4.3 Microbial network restructuring and climate feedback regulation

Beyond shifts in specific functional microbial guilds, long-term organic farming significantly altered the structure of cross-domain microbial ecological networks, particularly those linking bacterial and fungal communities. These network-level changes provide mechanistic support for our third hypothesis that organic management reshapes microbial network topology by modulating keystone species abundance, thereby influencing soil functionality and GHG emissions.

Microbial network complexity is widely recognized as an important regulator of ecosystem multifunctionality and stability [67,68]. However, our results revealed contrasting patterns under different seasonal contexts: organic farming increases microbial co-occurrence network complexity in the upland season but decreases it in the rice season. Notably, these changes were accompanied by inverse responses in soil health, indicating that higher network complexity does not necessarily translate into improved soil functionality (Fig. 4). Several explanations may account for this pattern. In the upland season, the C-RW system experienced higher land-use intensity, including frequent tillage and pesticide application. These disturbances likely disrupted soil aggregate structure, reduced porosity and water retention, and fragmented microbial habitats [69,70], thereby weakening bacterial-fungal interactions and simplifying microbial connectivity. At the same time, elevated nutrient availability under C-RW, characterized by higher mineral N and lower C/N ratio, may have temporarily supported soil functioning despite reduced network integrity [17]. Additionally, microbial interaction networks are inherently sensitive to spatiotemporal variability [71], which may further modulate ecosystem responses to management practices. This pronounced seasonal plasticity in microbial network architecture, shifting between a more complex state in the upland season and a simplified state in the flooded rice season, may be a key mechanistic adaptation of microorganisms to environmental changes under the organic farming system. It demonstrates the system’s capacity to dynamically reconfigure microbial interactions in response to radically different hydrological and physicochemical conditions, thereby maintaining soil health and function across seasons.

In the rice season, microbial networks under C-RW exhibited higher complexity than those under O-RD, yet soil health indices were significantly lower in C-RW. Although higher microbial network complexity is often associated with greater ecosystem stability and health [72,73], an increasing body of evidence suggests that this relationship can be weak, non-linear, or even negative in agricultural systems. Agricultural disturbance may decouple microbial network complexity from soil functioning by disrupting long-term established interactions [54,74]. In this context, the lower microbial network complexity observed under long-term organic management may reflect a community that has been selectively shaped over 15 years to efficiently process recurring organic inputs. Reduced network complexity may indicate the loss of redundant or non-essential interactions, allowing for more direct and efficient carbon and nutrient flows [75]. This discrepancy may be further attributed to the significantly higher fungal diversity observed under organic management (*P* < 0.001; Fig. S7g). Enhanced fungal diversity represents a fundamental shift in the soil’s ‘biological engine’ toward improved carbon processing efficiency, stronger soil structure formation, and more integrated plant-microbe interactions. Together, these processes underpin the enhanced soil health and sustainability of the organic system [76]. Consequently, the positive impact of biodiversity enrichment under organic farming may decouple the typically assumed linkage between microbial network complexity and soil functionality [77,78]. These findings suggest that evaluating soil health solely based on microbial network complexity may be misleading, and that land-use intensity, microbial diversity, and disturbance regimes must be considered simultaneously.

Consistent with our third hypothesis, the abundance of key species in microbial networks was an important factor influencing GHG emissions. In the upland season, the ratio of (AOA + AOB + *nir*K + *nir*S)/*nos*Z was positively correlated with the keystone species abundance, while in the rice season, methanotroph abundance showed a negative correlation with keystone species (Fig. 5b, d). These relationships suggest that organic farming may indirectly reduce N_2_O and CH_4_ emissions by regulating the abundance of keystone species (Fig. 5b, d). This interpretation aligns with previous studies showing that changes in the abundance of key species, such as *Deinococcus-Thermus* and *Gemmatimonadetes* phylum, can substantially affect N_2_O emissions [79]. Changes in keystone species abundance can therefore influence the balance of N-cycling functional guilds and modulate GHG production pathways [80,81]. Our results further suggest that the abundance of methanogens in the rice season was directly and negatively associated with keystone species abundance. One plausible explanation is that certain keystone taxa, such as *Actinobacteriota*, participate in C and N metabolism in ways that reduce substrate availability for methanogens or methanotrophs [82]. Through such interactions, long-term organic management may indirectly regulate CH_4_ dynamics by reshaping microbial interaction hierarchies rather than by directly suppressing methanogenic potential.

Overall, this study provided a general picture for understanding the effects of organic farming on soil health and GHG emission potential (Fig. 6). Based on evidence from a long-term experimental site, our results demonstrate that organic management with rice-duck co-culture can enhance soil health, reshape microbial communities toward a climate-mitigating state, and improve economic viability. Although these findings are context-specific, they offer valuable insights and a validated conceptual framework for sustainable agricultural practices. The observed synergy between ecological and economic benefits highlights a promising pathway for advancing agricultural sustainability. However, it should be noted that the GHG fluxes reported here were measured under controlled laboratory incubations and thus represent the potential activity of microbial communities under standardized temperature and moisture conditions. Besides, the 2-week incubation period, while commonly used and often necessary to achieve sufficient ^13^C incorporation into DNA, carries an inherent risk of inducing secondary microbial community shifts and cross-feeding effects, whereby initially labeled organisms are consumed by secondary feeders. Therefore, future research across diverse agroecosystems is needed to test the generalizability of these results under field conditions. Nonetheless, this work provides critical mechanistic evidence and a conceptual framework that can inform the design of management strategies aimed at developing resilient and profitable agroecosystems.

**Fig. 6:**
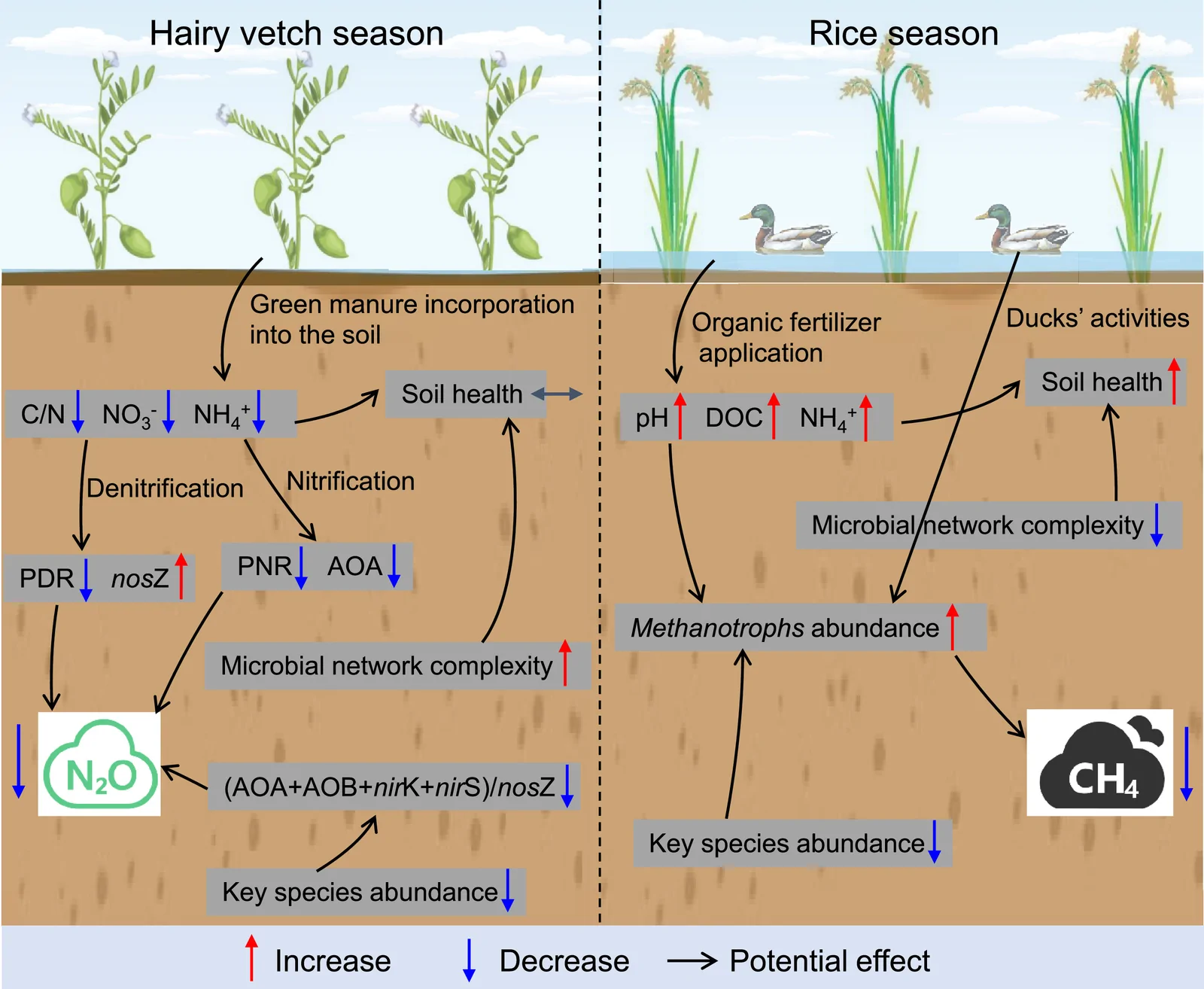
**Schematic illustration of system integration from issues to outcomes in organic rice-vetch rotation with rice-duck co-culture.** The diagram links management practices across the hairy vetch and rice seasons, highlighting interactions among N transformations, microbial networks, and soil health. Red upward arrows indicate variables or processes that increase under O-RD relative to the conventional rice-wheat rotation, whereas blue downward arrows denote those that decrease. Black arrows represent potential causal or functional influences among processes.

## CRediT authorship contribution statement

**Zhaoqiang Han:** Writing – original draft, Software, Methodology, Investigation, Funding acquisition, Formal analysis, Data curation. **Ziheng Zou:** Writing – original draft, Methodology, Investigation, Formal analysis. **Yubing Dong:** Writing – review & editing, Resources. **Yi Leng:** Writing – review & editing, Investigation. **Shumin Guo:** Writing – review & editing, Methodology. **Zhe Xu:** Writing – review & editing, Investigation. **Longping Tu:** Writing – review & editing, Investigation. **Huacheng Yin:** Investigation. **Yang Ruan:** Writing – review & editing, Methodology. **Shuwei Liu:** Writing – review & editing. **Jinyang Wang:** Writing – review & editing, Supervision, Project administration, Conceptualization. **Jianwen Zou:** Writing – review & editing, Supervision, Conceptualization.

## Declaration of competing interest

The authors declare that they have no conflicts of interest in this work.

## Appendix Supplementary materials

Image, application 1
