# Supplementary material for: Enhanced soil health and greenhouse gas mitigation potential are linked to microbial network restructuring under long-term organic farming
Source: Fundam Res. 2026 Apr 23;6(5):2986–98. doi: 10.1016/j.fmre.2026.04.014 (PMC13644493; doi:10.1016/j.fmre.2026.04.014)
Supplement: Supplementary file 1 [file mmc1.docx]

**Supplementary Materials for**

**Enhanced soil health and greenhouse gas mitigation potential are linked to microbial network restructuring under long-term organic farming**

**Supplementary Methods**

**Soil health assessment**

The Cornell Soil Health Assessment (CSHA) scoring method was used to calculate the soil health score (Teng et al., 2024). Fourteen soil properties that are important to soil productivity, nutrient cycling, and stability, including soil pH, total N, total C, C/N ratio, NH_4_^+^, NO_3_^-^, DOC, BG, NAG, AP, LAP, PPO, PER, and urease were normalized as individual CSHA scores (Yang et al., 2024). The weight value of individual soil indicators was based on PCA of all soil indicators, representing the sum of the eigenvectors derived from the first three principal components, which were selected based on Kaiser’s cut-off (eigenvalues > 1). These first three principal components cumulatively accounted for over 80% and 87% of the variance in the upland and rice seasons, respectively, capturing most of the variation among the soil indicators. The overall soil health score (%) was computed as a weighted average of all individual CSHA scores as follows:

$soil health score= \frac{A_{1}\times w_{1}+A_{2}\times w_{2}+\cdots+A_{n}\times w_{n}}{w_{1}+w_{2}+\cdots+w_{n}}$	(9)

where A is the CSHA score for each individual soil indicator, and w is the weighting value of soil indicators (see Fig. S2c and d).

**N_2_O and CH_4_ emissions fluxes**

Before each gas sample collection, each flask was sealed with a rubber stopper with a three-way valve, and 15 mL of gas was collected at 0 and 2 hours after sealing. The concentrations of N_2_O and CH_4_ were analyzed using a modified gas chromatograph (Agilent 7890B) equipped with electron capture and flame ionization detectors. Both N_2_O and CH_4_ fluxes were calculated according to the following equation:

$F=\left( \rho\times v/w \right)\times\left( \Delta C/\Delta t \right)\times\left[ \left( 273/\left( 273+T \right) \right) \right]$	(1)

where $F$ is N_2_O or CH_4_ flux (μg kg^-1^ h^-1^); ρ is the density of N_2_O or CH_4_ (kg m^-3^) at standard pressure and temperature; $v$ is the volume of flask (m^-3^); $w$ is the weigh of soils (0.05 kg); $\Delta C/\Delta t$is the gas concentration change between 0 and 2 hours (ppm h^-1^); $T$is the temperature inside the flask (^o^C). The daily mean flux of N_2_O or CH_4_ for the sampling day is represented by flux over the two-hour sampling period. The cumulative N_2_O or CH_2_ emissions were determined by accumulating fluxes sequentially between every two adjacent sampling days during the whole incubation period (Zou et al., 2005).

**Soil potential nitrification (PNR) and denitrification rates (PDR)**

For determining the PNR, three portions of fresh soil (3 g dry mass) of each soil sample were weighed and filled into three 120 ml serum bottles, respectively. Incorporated 20 ml of substrate solution containing 0.2 M K_2_HPO_4_, 0.2 M KH_2_PO_4_, and 0.02 M NH_4_SO_4_ into each serum bottle (Norton & Stark, 2011). The soil and solution in the bottle were vortexed and broken up into a slurry, and shaken aerobically at 200 r/min for 2 h at 25°C. The serum bottles were sealed with rubber stoppers and 5 mL of gas samples were collected. No extra gas, 288 μl C_2_H_2_ (10% *v*/*v*), and 2.592 ml 1-octyne (0.03% *v*/*v*) were respectively injected into three serum bottles, which were labeled as A, B, and C (Mushinski et al., 2019). All serum bottles were continued shaken at 200 r/min for 22 h at 25°C, and then another 5 mL of gas samples were collected. Determined the N_2_O concentration of gas samples taken at 2 h and 22 h by gas chromatography and calculated the N_2_O flux according to the method in 2.3. Total nitrification rate, heterotrophic nitrification rate, autotrophic nitrification dominated by AOA-*amo*A, and autotrophic nitrification dominated by AOB-*amo*A were represented by the N_2_O flux in A, B, C-B, and A-C, respectively.

For determining soil PDR, 3 g of fresh soil (dry mass) of each soil sample was weighed and filled into 25 ml serum bottles. The soil and solution in the bottle were vortexed and broken up into a slurry after being sealed with rubber stoppers. The gas pressure in the bottle was pumped to less than 0.1 atm with a vacuum pump, followed by an injection of 25 mL of C_2_H_2_ (10% *v*/*v*) to inhibit the reduction of N_2_O to N_2_. After incubation at 25°C for 3 h, 5 mL of the gas in the bottle was withdrawn, and the N_2_O concentration was determined by gas chromatography according to the method in 2.3. The PDR (μg kg^-1^ h^-1^) in each serum bottle was calculated by dividing the concentration of N_2_O produced in 3 h by the incubation time.

**Isotopic analysis and calculation of N_2_O production and consumption**

The net isotope effect, the difference in isotope values between the product and substrate, is commonly applied to isotope identification depending on the premise that most biological reactions involve multiple fractionation steps (Lewicka-Szczebak et al., 2020). In the study, stable isotope ratios of nitrogen (δ^15^N) and oxygen (δ^18^O), and N_2_O isotopomers (δ^15^N^α^ and δ^15^N^β^) were determined by using a gas chromatograph-isotope ratio mass spectrometer (GC-IRMS, Thermo Fisher Scientific [China] Co., Ltd., Beijing, China). The *δ*^15^N^bulk^, *δ*^18^O_N2O_, *δ*^15^N_N2O_, and site preference (SP) values were calculated using the following equations (Toyoda et al., 2005):

*δ*^15^N*^i^ =* ^15^$R_{sample}^{i}$ */* ^15^$R_{standard}$ – 1 (i=α, β and bulk)	(2)

*δ*^18^O *=*^18^$R_{sample}$ / $R_{standard}$ – 1	(3)

*δ*^15^N^bulk^ = (*δ*^15^N*^α^ + δ*^15^N*^β^*) / 2	(4)

SP = *δ*^15^N*^α^* – *δ*^15^N*^β^*	(5)

where R is the mean isotope ratio for ^15^N/^14^N or ^18^O/^16^O, which are given in ‰ deviation from the international standards of ^15^N and ^18^O are atmospheric N_2_ and Vienna Standard Mean Ocean Water (V-SMOW), respectively. The difference between *δ*^15^N*^α^* and *δ*^15^N*^β^* is referred to as the ^15^N site preference (SP, in ‰).

The mapping approach model of *δ*^15^N^SP^ − *δ*^18^O_N2O_ (SP/O MAP) was to estimate the contribution of various pathways to N_2_O production and consumption. The two-pool model was used to calculate isotopic values of soil air, respectively (Yano et al., 2014):

$$\delta_{N_{2}O[soil]}=\frac{((\delta_{N_{2}O\left[ concentration \right]}*c_{N_{2}O\left[ concentration \right]})-(\delta_{N_{2}O\left[ background \right]}*c_{N_{2}O\left[ background \right]}))}{(c_{N_{2}O\left[ concentration \right]}-c_{N_{2}O\left[ background \right]})}$$

(6)

where $c_{N_{2}O\left[ concentration \right]}$ and $c_{N_{2}O\left[ background \right]}$ denote concentrations of N_2_O gas sampled from PVC soil-air equilibration sampler and ambient field air, respectively. Values for δ^15^N^SP^ of background air were 18.0‰.

The SP value of the produced N_2_O (SP_0_) was calculated using a Rayleigh-type model, assuming that isotope dynamics followed closed-system behavior (Lewicka-Szczebak et al., 2014). The model can be described as follows:

$\mathrm{SP}_{N_{2}O-r}$ = $\mathrm{SP}_{0}$+ *η*_r_$\ln\left( \frac{C}{C_{0}} \right)$	(7)

In this equation, $\mathrm{SP}_{N_{2}O-r}$ is the SP value of the remaining N_2_O, $\mathrm{SP}_{0}$ is the SP value of the initial N_2_O, *η*_r_ is the net isotope effect (NIE) associated with N_2_O reduction, and $C$ and $C_{0}$ are the residual and the initial N_2_O concentration (i.e. $\frac{C}{C_{0}}$ expresses the N_2_O/(N_2_O+N_2_) product ratio). As measuring *in-situ* N_2_ emission is still impossible, the mapping approach model using parameters based on the weighted median values reported in the current literature was used to quantify the N_2_O/(N_2_O+N_2_) product ratio (Wu et al., 2019). The contribution of different microbial processes to N_2_O production is calculated according to the equation below:

$\mathrm{SP}_{0}$ = $\mathrm{SP}_{D}$ ∙ f_D-SP_ + SP_N_ ∙ f_N-SP_	(8)

In this equation, f_D-SP_ and f_N-SP_ represent the contribution of denitrification and nitrification/fungal denitrification to total N_2_O production based on the SP values, respectively. In this equation it is assumed that we deal with only two end-members, hence f_N-SP_ + f_D-SP_ = 1. Since fungal denitrification is known to have similar SP values as nitrification, distinguishing N_2_O originating from fungal denitrification and bacterial nitrification based on SP values is mathematically impossible. Therefore, the isotopic signatures of the end members were defined as 37‰ for both bacterial nitrification and fungal denitrification, and -5‰ for bacterial denitrification and nitrifier denitrification (Toyoda et al., 2005).

**N_2_O isotope mapping approach**

The mapping approach is based on the different slopes of the mixing line between *bD* (possibly including also *nD*) and *fD* or *Ni* and the reduction line reflecting isotopic enrichment of residual N_2_O due to its partial reduction in dual-isotope plots. Both lines are defined from the known most relevant literature data on the respective mixing endmember isotopic signatures and reduction fractionation factors. The detailed isotopic characteristics applied for the isotope MAPs are presented in Table S5 and follow the most recent review paper (Yu et al., 2020). The calculations are performed according to two possible cases of N_2_O mixing and reduction:

Case 1 – N_2_O produced from *bD* is first partially reduced to N_2_, followed by mixing of the residual N_2_O with N_2_O from other pathways.

Case 2 – N_2_O produced by various pathways is first mixed and afterwards reduced.

The calculations can be performed following different scenarios of particular endmember mixing: either *bD*-*fD* mixing or *bD*-*Ni* mixing. The detailed information for the cases calculation can be found in the recent study (Lewicka-Szczebak et al., 2020).

**Soil CH_4_ production and oxidation potentials**

For measuring soil CH_4_ production potential, 2.5 g dry weight of fresh soil was weighed and spread flat on the bottom of a 25 mL serum bottle, 5 mL of deionized water was added to provide a 1 cm water layer on top of the soil, and the bottle was sealed with a rubber stopper. After pumping the air pressure in the bottle to below 0.1 atm with a vacuum pump, 25 mL of high-purity nitrogen gas was added. The treated samples were placed in an incubator at 25°C, and 2 mL of gas was collected at the 2nd, 48th, and 72nd h. After each gas extraction, the extracted gas samples were diluted twice with N_2_, and 2 mL of N_2_ was injected into the serum bottles to maintain the pressure equilibrium. The CH_4_ concentration at the three sampling nodes was determined by gas chromatography, and the amount of CH_4_ produced per unit of time was calculated by linear fitting as the CH_4_ production potential (μg kg^-1^ h^-1^) (Mo et al., 2025).

The soil CH_4_ oxidation potential was characterized by the amount of methane oxidized per unit time of soil under methane-saturated conditions. The experimental detail was as follows: 10 g of fresh soil was added to a 150 mL serum bottle, which was sealed with a rubber stopper after adding 10 mL of pure water. The bottle was filled with 1.5 mL of methane (1%, 10000 ppmv), placed on a shaker, and incubated for 2 h at a constant temperature of 25 °C, protected from light, and then 2 mL of gas was taken as the initial concentration of methane. Then 2 mL of gas was collected after the 12th, 24th, and 48th h, respectively. After each pumping, the collected gas samples were diluted with 2 mL of nitrogen, and 2 mL of air was injected into the serum vials to maintain the pressure equilibrium in the serum vials. The CH_4_ concentration at each sampling node was determined by gas chromatography, and the amount of CH_4_ oxidized per unit time was calculated by linear fitting as methane oxidation potential (μg kg^-1^ h^-1^) (Mo et al., 2025).

**Fig. S1 The CH_4_ concentrations during the respective DNA-SIP incubation**. **a-b** The CH_4_ concentrations under glucose-labeled to investigate methanogens abundance. **c-d** The CH_4_ concentrations under CH_4_-labeled to investigate methanotrophs abundance.

**Fig. S2 a-b** Principal component analyses (PCA) for fourteen soil properties. **c-d** The weight value of each soil property, representing the sum of the eigenvectors derived from the first three principal components. Treatment abbreviations: C-RW conventional rice-wheat rotation, O-RD rice-hairy vetch rotation combined with rice-duck farming.

**Fig. S3** CO_2_ emission potential during the upland (a) and rice (b) seasons, respectively. The cumulative CO_2_ emissions during the whole incubation experiment (c).

**Fig. S4** Economic benefit for the two farming systems. Treatment abbreviations: C-RW conventional rice-wheat rotation, O-RD organic rice-vetch rotation combined with rice-duck co-culture

**Fig. S5** The difference in N-cycling genes abundance (copy numbers (log-10-transformed) per g dry soil) across the two cropping patterns. The solid black ball represents corresponding data points for the bar chart, *n* = 5. The black square boxes and solid lines inside the boxes are the mean and median values for different treatments, respectively. Box boundaries represent the 75th and 25th percentiles, whisker caps represent the 95th and 5th percentiles. The results of “*”, “**”, and “***” represent *P* < 0.05, *P* < 0.01, and *P* < 0.001, respectively, and “ns” represents there is no significant difference between the two systems.

**Fig. S6** The abundance of *mcr*A, *pmo*A, and the ratio of *mcr*A/*pmo*A (copy numbers (log-10-transformed) per g dry soil) in different cropping patterns. The solid black ball represents corresponding data points for the bar chart, *n* = 5. The black square boxes and solid lines inside the boxes are the mean and median values for different treatments, respectively. Box boundaries represent the 75th and 25th percentiles, whisker caps represent the 95th and 5th percentiles. The results of “*”, “**”, and “***” represent *P* < 0.05, *P* < 0.01, and *P* < 0.001, respectively, and “ns” represents there is no significant difference between the two systems.

**Fig. S7 Microbial diversity and community structure in different cropping patterns. a-b** Bacterial Shannon index and PCoA analyses in upland season. **c-d F**ungal Shannon index and PCoA analyses in upland season. **e-f** Bacterial Shannon index and PCoA analyses in rice season. **g-h** Fungal Shannon index and PCoA analyses in upland season. **i-j** the relative abundance of the dominant bacterial and fungal phyla in upland and rice season, respectively. In **a, c, e,** and **g**, the whiskers in each column indicate the standard deviation of five replications.

**Fig. S8 Zi-Pi plots and key species abundance in microbial co-occurrence networks.**

**a-c** Zi-Pi plots, and relative abundance of key species in the upland season. **d-f** Zi-Pi plots, and relative abundance of key species in the rice season.

Table S1 Annual agricultural inputs for each agricultural system in 2022

| Systems | Crop/input^a^ | COF^b^  (kg N ha^-1^) | Urea  (kg N ha^-1^) | RCF^c^  (kg N ha^-1^) | OF^d^  (kg N ha^-1^) | P_2_O5  (kg ha^-1^) | K_2_O  (kg ha^-1^) | Diesel  (kg ha^-1^) | Electricity^e^  (kWh ha^-1^) | Pesticide^f^  (kg ha^-1^) | Seed  (kg ha^-1^) | Labor  (h ha^-1^) | Duckling | Forage (kg ha^-1^) |
| --- | --- | --- | --- | --- | --- | --- | --- | --- | --- | --- | --- | --- | --- | --- |
| C-RW | Wheat | 101.3 | 167.9 | 0 | 0 | 101.3 | 101.3 | 63.8 | 0 | 15.8 | 375 | 236 | 0 | 0 |
|  | Wheat-input  (Yuan ha^-1^) | 675.3 | 772.3 | 0 | 0 | 675.3 | 675.3 | 523.8 | 0 | 1343 | 3000 | 3540 | 0 | 0 |
|  | Rice | 105.3 | 157.7 | 0 | 0 | 105.3 | 105.3 | 89.3 | 823.5 | 16.6 | 55 | 296 | 0 | 0 |
|  | Rice-input  (Yuan ha^-1^) | 702 | 725.4 | 0 | 0 | 702 | 702 | 732.3 | 411.8 | 1411 | 1100 | 4440 | 0 | 0 |
| O-RD | Vetch | 0 | 0 | 0 | 0 | 0 | 0 | 51 | 0 | 10.2 | 75 | 56 | 0 | 0 |
|  | Vetch-input  (Yuan ha^-1^) | 0 | 0 | 0 | 0 | 0 | 0 | 418.2 | 0 | 867 | 1125 | 840 | 0 | 0 |
|  | Rice | 0 | 0 | 139.2 | 112.5 | 0 | 0 | 89.3 | 936.5 | 0 | 55 | 388 | 225 | 1800 |
|  | Rice-input  (Yuan ha^-1^) | 0 | 0 | 7920 | 9000 | 0 | 0 | 732.3 | 468.3 | 0 | 1100 | 5820 | 337.5 | 2700 |

^a^Cost input is based on the actual field situation, including seed, fertilizer, electricity for irrigation, and fuel cost.

^b^COF: Compound fertilizer; ^c^RCF: Rapeseed cake fertilizer; ^d^OF: Organic fertilizer.

^e^Electricity for irrigation is calculated as crop irrigation consumption (m^3^ ha^–1^) × unit electricity consumption (kWh m^–3^), and the unit for electricity consumption is 0.4 kWh m^–3^.

^f^Pesticide values are the sum of the active ingredients for herbicides, insecticides, and fungicides.

**Table S2** The yields and corresponding gross benefits of wheat, rice and ducks in 2022

| Systems | Items | Yield  (kg ha^-1^) | Gross benefit  (Yuan ha^-1^) |
| --- | --- | --- | --- |
| C-RW | Wheat | 9442.5 | 24361.7 |
|  | Rice | 11284.5 | 29339.7 |
| O-RD | Vetch | - | - |
|  | Rice | 7477.5 | 119640 |
|  | Duck | 450 | 22500 |

**Table S3** The individual soil functions used for calculating the effect sizes of soil multifunctionality.

| Soil functions | Units | Description |
| --- | --- | --- |
| pH | - | Soil pH plays a significant role in influencing nutrient availability and microbial activity (Mitsuta et al., 2025). |
| Total C | g/kg | SOC usually interacts with many biotic and abiotic components of ecosystems and plays important role in regulating climate. |
| Total N | g/kg | Build-up of nutrient pools that most frequently limit the primary production in diverse ecosystems. |
| C/N | - | An appropriate C/N ratio can promote the decomposition of organic matter and enhance soil fertility (Cui et al., 2022). |
| Available N | mg/kg | Play an important role in [terrestrial ecosystems](https://www.sciencedirect.com/topics/agricultural-and-biological-sciences/terrestrial-ecosystem) by affecting soil microbial movement, [litter decomposition](https://www.sciencedirect.com/topics/agricultural-and-biological-sciences/litter-decomposition), and nutrients cycling and accumulation (Griffiths et al., 2009). |
| DOC | mg/kg | DOC represents an important portion of terrestrial organic matter, playing a role in mediating energy and nutrient transfer between land, water, and the atmosphere (Battin et al., 2009). |
| EEA | - | Involved in the synthesis and decomposition of humus in soil, hydrolysis and transformation of organic compounds, plant, animal and microbial residues, and various REDOX reactions of organic and inorganic compounds in soil. |

**Table S4** Gene-specific primers and thermal cycling conditions followed established protocols in this study

| Gene | Name | Sequence | Thermal profile | No. cycles | Product size | Reference | |  |
| --- | --- | --- | --- | --- | --- | --- | --- | --- |
| AOA *amoA* | CrenamoA23f | ATGGTCTGGCTWAGACG | 30 s-95°C, 95°C-15 s, 55°C-30 s,72°C-30 s,  80°C-30 s | 1 | 635bp | (Nicol et al., 2008) | |  |
|  | CrenamoA616r | GCCATCCATCTGTATGTCCA | 95°C-5 s, 57°C-34 s, 72°C-15 s 95°C-15 s,  55°C-30 s,72°C-30 s, 80°C-30 s | 40 |  |  | |  |
| AOB *amoA* | *amoA*-1F | GGGGTTTCTACTGGTGGT | 30 s-95°C,95°C-15 s, 55°C-30 s,72°C-30 s,  80°C-30 s | 1 | 491bp | (Rotthauwe et al., 1997) | |  |
|  | *amoA*-2R | CCCCTCKGSAAAGCCTTCTTC | 95°C-5 s, 55°C-34 s, 72°C-15 s 95°C-15 s,  55°C-30 s, 72°C-30 s, 80°C-30 s | 40 |  |  | |  |
| *nirK* | *nirK*-F1aCu | ATCATGGTSCTGCCGCG | 30 s-95°C,95°C-15 s, 55°C-30 s,72°C-30 s,  80°C-30 s | 1 | 473bp | (Throbäck et al., 2004) | |  |
|  | *nirK*-R3Cu | GCCTCGATCAGRTTGTGGTT | 95°C-5 s, 58°C-34 s, 72°C-15 s 95°C-15 s,  55°C-30 s,72°C-30 s, 80°C-30 s | 40 |  |  | |  |
| *nirS* | *nirS*-Cd3aF | TACCACCCSGARCCGCGCGT | 30 s-95°C, 95°C-15 s, 55°C-30 s,72°C-30 s,  80°C-30 s | 1 | 425bp | (Throbäck et al., 2004) | |  |
|  | *nirS*-R3cd | GCCGCCGTCRTGVAGGAA | 95°C-5 s, 58°C-34 s, 72°C-15 s 95°C-15 s,  55°C-30 s,72°C-30 s, 80°C-30 s | 40 |  |  | |  |
| *nosZ* | *nosZ*-F | AGAACGACCAGCTGATCGACA | 30 s-95°C, 95°C-15 s, 55°C-30 s,72°C-30 s,  80°C-30 s | 1 | 300bp | (Scala & Kerkhof, 1998) | |  |
|  | *nosZ*-R | TCCATGGTGACGCCGTGGTTG | 95°C-5 s, 60°C-34 s, 72°C-15 s 95°C-15 s,  55°C-30 s, 72°C-30 s, 80°C-30 s | 40 |  | |  | |
| *mcr*A | mcrA-R | GGTGGTGTMGGATTCA | 30 s-95 ^o^C, 95 ^o^C -15 s, 55 ^o^C -30 s,72 ^o^C -30 s, 80 ^o^C -30 s | 1 | 509 bp | | (Steinberg & Regan, 2009) | |
|  | mcrA-F | CACARTAYGCWACAGC | 95 ^o^C -5 s, 60 ^o^C -34 s, 72 ^o^C -15 s 95 ^o^C -15 s, 55 ^o^C -30 s,72 ^o^C -30 s, 80 ^o^C -30 s | 40 |  | |  | |
| *pmo*A | pmoA-R | GGNGACTGGGACTTCTGG | 30 s-95 ^o^C, 95 ^o^C -15 s, 55 ^o^C -30 s,72 ^o^C -30 s, 80 ^o^C -30 s | 1 | 472 bp | | (Costello & Lidstrom, 1999) | |
|  | pmoA-F | CCGGMGCAACGTCYTTACC | 95 ^o^C -5 s, 60 ^o^C -34 s, 72 ^o^C -15 s 95 ^o^C -15 s, 55 ^o^C -30 s,72 ^o^C -30 s, 80 ^o^C -30 s | 40 |  | |  | |

**Table S5** The discrepancy of different soil properties across three cropping patterns

| Season | Soil properties | C-RW | O-RD |
| --- | --- | --- | --- |
| Upland | pH | 4.84±0.05b | 5.08±0.06a |
|  | Total C (g kg^-1^) | 24.38±1.08a | 23.29±4.59a |
|  | Total N (g kg^-1^) | 2.64±0.22a | 1.97±0.36b |
|  | C/N | 9.28±0.78b | 11.78±0.34a |
|  | NH_4_^+^ (mg kg^-1^) | 65.56±56.45a | 18.81±4.19a |
|  | NO_3_^–^ (mg kg^-1^) | 198.02±21.66a | 96.28±11.72b |
|  | DOC (mg kg^-1^) | 195.39±37.14b | 253.09±28.79a |
|  | BG (μmol h^-1^ g^-1^) | 0.023±0.001b | 0.026±0.002a |
|  | NAG (μmol h^-1^ g^-1^) | 0.015±0.004a | 0.011±0.002a |
|  | AP (μmol h^-1^ g^-1^) | 0.068±0.002a | 0.165±0.18a |
|  | LAP (μmol h^-1^ g^-1^) | 0.056±0.012a | 0.025±0.006b |
|  | PPO (μmol h^-1^ g^-1^) | 0.19±0.037a | 0.18±0.044a |
|  | PER (μmol h^-1^ g^-1^) | 1.86±0.22a | 1.09±0.17b |
|  | Urease (mg h^-1^ g^-1^) | 0.85±0.064b | 0.95±0.052a |
| Rice | pH | 5.17±0.09b | 5.72±0.10a |
|  | Total C (g kg^-1^) | 25.47±0.66b | 31.15±3.55a |
|  | Total N (g kg^-1^) | 2.18±0.16a | 2.55±0.48a |
|  | C/N | 11.71±0.60a | 12.60±2.69a |
|  | NH_4_^+^ (mg kg^-1^) | 142.54±20.17b | 214.75±23.13a |
|  | NO_3_^–^ (mg kg^-1^) | 4.35±0.92a | 2.06±0.71b |
|  | DOC (mg kg^-1^) | 141.77±33.02b | 211.29±30.68a |
|  | BG (μmol h^-1^ g^-1^) | 0.014±0.002b | 0.023±0.006a |
|  | NAG (μmol h^-1^ g^-1^) | 0.059±0.009a | 0.056±0.010a |
|  | AP (μmol h^-1^ g^-1^) | 0.14±0.034a | 0.11±0.021a |
|  | LAP (μmol h^-1^ g^-1^) | 0.014±0.004b | 0.022±0.004a |
|  | PPO (μmol h^-1^ g^-1^) | 0.018±0.004b | 0.023±0.003a |
|  | PER (μmol h^-1^ g^-1^) | 0.019±0.005b | 0.049±0.007a |
|  | Urease (mg h^-1^ g^-1^) | 0.73±0.03b | 0.81±0.02a |

**Note:** C-RW traditional rice-wheat rotation, O-RD rice-hairy vetch rotation combined with rice-duck farming. Different letters following numbers indicate significant differences at the *P* < 0.05 level.

**Table S6** Range of δ^15^N, δ^18^O, and δ^15^N^SP^ for each microbial proco-25.3ess obtained in pure culture assays. All the data was from summaries by previous studies (**Wu et al., 2019; Yu et al., 2020**).

| Microbial processes | Range of N_2_O isotope values | | | | | |
| --- | --- | --- | --- | --- | --- | --- |
|  | δ^15^N^bulk^ (‰) | | δ^18^O (‰) | | δ^15^N^SP^ (‰) | |
|  | Min | Max | Min | Max | Min | Max |
| Bacterial denitrification (*bD*) | -52.8 | 2.3 | 4.5 | 46.5 | -8.7 | 8.5 |
| Nitrifier denitrification (*nD*) | -60.7 | -53.1 | 12.4 | 19.4 | -13.6 | 1.9 |
| Nitrification (*Ni*) | -64.0 | -47.0 | 19.3 | 65.0 | 9.2 | 40.0 |
| Fungal denitrification (*fD*) | -46.0 | -31.0 | 19.3 | 65.0 | 9.2 | 40.0 |

**Table S7** Quantitative distribution of *pmo*A gene copy numbers across the buoyant density gradients of the fractionated DNA

| Microbes | Treatment | Fraction | CsCl buoyant density  (g mL^-1^) | *pmoa*A gene abundance  (log copies g^-1^) |
| --- | --- | --- | --- | --- |
| *methanotrophs* | RW-^12^C | F6 | 1.729 | 1.412 |
|  |  | F7 | 1.726 | 1.968 |
|  |  | F8 | 1.722 | 3.264 |
|  | RW-^13^C | F6 | 1.73 | 4.379 |
|  |  | F7 | 1.727 | 4.103 |
|  |  | F8 | 1.722 | 3.473 |
|  | RD-^12^C | F6 | 1.732 | 1.56 |
|  |  | F7 | 1.729 | 2.018 |
|  |  | F8 | 1.724 | 2.853 |
|  | RD-^13^C | F6 | 1.728 | 2.671 |
|  |  | F7 | 1.725 | 3.74 |
|  |  | F8 | 1.721 | 3.91 |
| *methanogens* | RW-^12^C | F4 | 1.735 | 1.244 |
|  |  | F5 | 1.731 | 1.203 |
|  |  | F7 | 1.722 | 1.435 |
|  | RW-^13^C | F4 | 1.736 | 1.632 |
|  |  | F5 | 1.732 | 1.64 |
|  |  | F7 | 1.725 | 1.864 |
|  | RD-^12^C | F4 | 1.731 | 1.047 |
|  |  | F5 | 1.727 | 0.691 |
|  |  | F7 | 1.719 | 1.181 |
|  |  | F8 | 1.715 | 1.76 |
|  | RD-^13^C | F4 | 1.732 | 1.7 |
|  |  | F5 | 1.729 | 1.379 |
|  |  | F7 | 1.722 | 1.959 |
|  |  | F8 | 1.716 | 2.437 |

**Table S8** Sequencing proportions for labeled taxa

| Microbes | Treatment | Total sequence number | *methanotrophs* sequence number | *methanotrophs* sequence frequency (%) |
| --- | --- | --- | --- | --- |
| *methanotrophs* | RW-^13^C | 23591 | 3813 | 16 |
|  | RD-^13^C | 23591 | 19762 | 84 |
| *methanogens* | RW-^13^C | 31644 | 7538 | 24 |
|  | RD-^13^C | 31644 | 7672 | 24 |

**Table S9** Bacterial and fungal keystone species in co-occurrence networks in upland season.

| Microorganism | ID | Generalists | Phylum | Class | Order | Family | Genus | Generalists |
| --- | --- | --- | --- | --- | --- | --- | --- | --- |
| Bacteria | OTU916 | Connectors | Firmicutes | Clostridia | Oscillospirales | Oscillospiraceae | Papillibacter | Unclassified |
|  | OTU180 | Connectors | Proteobacteria | Alphaproteobacteria | Azospirillales | Azospirillaceae | Skermanella | Unclassified |
|  | OTU373 | Connectors | Chloroflexi | Anaerolineae | Anaerolineales | Anaerolineaceae | Unclassified | Unclassified |
|  | OTU72 | Connectors | Actinobacteriota | Thermoleophilia | Gaiellales | Unclassified | Unclassified | Unclassified |
|  | OTU43 | Connectors | Chloroflexi | Ktedonobacteria | Ktedonobacterales | JG30-KF-AS9 | Unclassified | Unclassified |
|  | OTU256 | Connectors | Chloroflexi | Unclassified | Unclassified | Unclassified | Unclassified | Unclassified |
|  | OTU95 | Connectors | Chloroflexi | Anaerolineae | Anaerolineales | Anaerolineaceae | Anaerolinea | uncultured_Anaerolinea_sp. |
|  | OTU251 | Connectors | Actinobacteriota | Actinobacteria | Frankiales | Frankiaceae | Frankiaceae | Unclassified |
|  | OTU704 | Connectors | Actinobacteriota | Thermoleophilia | Thermoleophilia | Unclassified | Unclassified | Unclassified |
|  | OTU271 | Connectors | Firmicutes | Limnochordia | Unclassified | Unclassified | Unclassified | Unclassified |
| Fungi | OTU215 | Module hubs | Basidiomycota | Microbotryomycetes | Leucosporidiales | Leucosporidiaceae | Unclassified | Unclassified |
|  | OTU258 | Module hubs | Unclassified | Unclassified | Unclassified | Unclassified | Unclassified | Unclassified |
|  | OTU44 | Module hubs | Unclassified | Unclassified | Unclassified | Unclassified | Unclassified | Unclassified |
|  | OTU57 | Module hubs | Unclassified | Unclassified | Unclassified | Unclassified | Unclassified | Unclassified |
|  | OTU173 | Module hubs | Ascomycota | Sordariomycetes | Hypocreales | Hypocreaceae | Hypocrea | Unclassified |
|  | OTU28 | Module hubs | Ascomycota | Sordariomycetes | Microascales | Microascaceae | Cephalotrichum | Doratomyces_nanus |
|  | OTU90 | Module hubs | Unclassified | Unclassified | Unclassified | Unclassified | Unclassified | Unclassified |
|  | OTU392 | Module hubs | Unclassified | Unclassified | Unclassified | Unclassified | Unclassified | Unclassified |
|  | OTU270 | Network hubs | Basidiomycota | Tremellomycetes | Tremellales | Tremellales_Incertae_sedis | Bullera | Bullera_pseudoalba |
|  | OTU29 | Module hubs | Ascomycota | Sordariomycetes | Hypocreales | Hypocreaceae | Hypocrea | Unclassified |
|  | OTU625 | Module hubs | Unclassified | Unclassified | Unclassified | Unclassified | Unclassified | Unclassified |
|  | OTU130 | Module hubs | Unclassified | Unclassified | Unclassified | Unclassified | Unclassified | Unclassified |
|  | OTU53 | Module hubs | Zygomycota | Mucoromycotina_Incertae_sedis | Mortierellales | Mortierellaceae | Mortierella | Mortierella_hyalina |
|  | OTU753 | Connectors | Unclassified | Unclassified | Unclassified | Unclassified | Unclassified | Unclassified |
|  | OTU183 | Connectors | Basidiomycota | Tremellomycetes | Tremellales | Tremellales_Incertae_sedis | Auriculibuller | Auriculibuller_fuscus |
|  | OTU38 | Connectors | Ascomycota | Unclassified | Unclassified | Unclassified | Unclassified | Unclassified |
|  | OTU264 | Connectors | Unclassified | Unclassified | Unclassified | Unclassified | Unclassified | Unclassified |
|  | OTU7 | Connectors | Unclassified | Unclassified | Unclassified | Unclassified | Unclassified | Unclassified |
|  | OTU18 | Connectors | Ascomycota | Sordariomycetes | Hypocreales | Hypocreales_Incertae_sedis | Villosiclava | Unclassified |
|  | OTU440 | Connectors | Unclassified | Unclassified | Unclassified | Unclassified | Unclassified | Unclassified |
|  | OTU211 | Connectors | Unclassified | Unclassified | Unclassified | Unclassified | Unclassified | Unclassified |

**Table S10** Bacterial and fungal keystone species in co-occurrence networks in rice season.

| Microorganism | ID | Generalists | Phylum | Class | Order | Family | Genus | Generalists |
| --- | --- | --- | --- | --- | --- | --- | --- | --- |
| Bacteria | OTU10 | Connectors | Firmicutes | Clostridia | Oscillospirales | Oscillospiraceae | Papillibacter | Unclassified |
|  | OTU813 | Connectors | Proteobacteria | Alphaproteobacteria | Azospirillales | Azospirillaceae | Skermanella | Unclassified |
|  | OTU76 | Connectors | Proteobacteria | Deltaproteobacteria | Unclassified | Unclassified | Unclassified | Unclassified |
|  | OTU150 | Connectors | Firmicutes | Clostridia | Clostridiales | Clostridiaceae | Clostridium_sensu_stricto_10 | Unclassified |
|  | OTU85 | Connectors | Actinobacteriota | Actinobacteria | Frankiales | Unclassified | Unclassified | Unclassified |
|  | OTU386 | Connectors | Firmicutes | Desulfitobacteriia | Desulfitobacteriales | Desulfitobacteriaceae | Desulfosporosinus | Unclassified |
|  | OTU289 | Connectors | Chloroflexi | Anaerolineae | SJA-15 | Unclassified | Unclassified | Unclassified |
|  | OTU59 | Connectors | Firmicutes | Clostridia | Peptostreptococcales-Tissierellales | Peptostreptococcaceae | Sporacetigenium | Unclassified |
|  | OTU420 | Module hubs | Firmicutes | Clostridia | Clostridiales | Clostridiaceae | Clostridium_sensu_stricto_10 | Unclassified |
|  | OTU529 | Connectors | Acidobacteriota | Vicinamibacteria | Vicinamibacterales | Unclassified | Unclassified | Unclassified |
|  | OTU299 | Connectors | Chloroflexi | Anaerolineae | Anaerolineales | Anaerolineaceae | Anaerolinea | Unclassified |
|  | OTU231 | Module hubs | Proteobacteria | Alphaproteobacteria | Caulobacterales | Caulobacteraceae | Phenylobacterium | uncultured_Phenylobacterium_sp. |
|  | OTU124 | Connectors | Firmicutes | Clostridia | Clostridiales | Clostridiaceae | Clostridium_sensu_stricto_1 | Unclassified |
|  | OTU86 | Connectors | Actinobacteriota | Actinobacteria | Propionibacteriales | Nocardioidaceae | Nocardioides | Unclassified |
|  | OTU757 | Connectors | Chloroflexi | Anaerolineae | Anaerolineales | Anaerolineaceae | Leptolinea | Unclassified |
|  | OTU806 | Connectors | Firmicutes | Clostridia | Clostridiales | Clostridiaceae | Clostridium | uncultured_Clostridium_sp. |
|  | OTU805 | Connectors | Firmicutes | Clostridia | Clostridiales | Caloramatoraceae | Fonticella | Unclassified |
|  | OTU277 | Connectors | Chloroflexi | Anaerolineae | Anaerolineales | Anaerolineaceae | Unclassified | Unclassified |
| Fungi | OTU276 | Connectors | Unclassified | Unclassified | Unclassified | Unclassified | Unclassified | Unclassified |
|  | OTU318 | Module hubs | Unclassified | Unclassified | Unclassified | Unclassified | Unclassified | Unclassified |
|  | OTU51 | Module hubs | Basidiomycota | Microbotryomycetes | Sporidiobolales | Sporidiobolales_Incertae_sedis | Sporobolomyces | Unclassified |
|  | OTU86 | Module hubs | Zygomycota | Mucoromycotina_Incertae_sedis | Mucorales | Mucoraceae | Absidia | Absidia_glauca |
|  | OTU596 | Module hubs | Unclassified | Unclassified | Unclassified | Unclassified | Unclassified | Unclassified |
|  | OTU151 | Connectors | Unclassified | Unclassified | Unclassified | Unclassified | Unclassified | Unclassified |
|  | OTU226 | Connectors | Ascomycota | Sordariomycetes | Melanosporales | Ceratostomataceae | Harzia | Acremonioides |
|  | OTU1 | Connectors | Unclassified | Unclassified | Unclassified | Unclassified | Unclassified | Unclassified |
|  | OTU492 | Module hubs | Unclassified | Unclassified | Unclassified | Unclassified | Unclassified | Unclassified |
|  | OTU389 | Connectors | Unclassified | Unclassified | Unclassified | Unclassified | Unclassified | Unclassified |
|  | OTU235 | Connectors | Ascomycota | Ascomycota_Incertae_sedis | Ascomycota_Incertae_sedis | Ascomycota_Incertae_sedis | Minutisphaera | Fimbriatispora |

Costello, A. M., & Lidstrom, M. E. (1999). Molecular characterization of functional and phylogenetic genes from natural populations of methanotrophs in lake sediments. *Applied and Environmental Microbiology*, *65*(11), 5066–5074. <https://doi.org/10.1128/aem.65.11.5066-5074.1999>

Cui, J., Zhu, R., Wang, X., Xu, X., Ai, C., He, P., Liang, G., Zhou, W., Zhu, P. (2022). Effect of high soil C/N ratio and nitrogen limitation caused by the long-term combined organic-inorganic fertilization on the soil microbial community structure and its dominated SOC decomposition. Journal of Environmental Management, 303, 114155. https://doi.org/10.1016/j.jenvman.2021.114155

Battin, T. J., Luyssaert, S., Kaplan, L. A., Aufdenkampe, A. K., Richter, A., Tranvik, L. J. 2009., The boundless carbon cycle. Nature geoscience, 2, 598-600. <https://doi.org/10.1038/ngeo618>

Griffiths, R. P., Madritch, M. D. & Swanson, A. K. (2009). The effects of topography on forest soil characteristics in the Oregon Cascade Mountains (USA): Implications for the effects of climate change on soil properties. Forest Ecology and Management, 257, 1–7. https://doi.org/10.1016/j.foreco.2008.08.010

Lewicka-Szczebak, D., Piotr Lewicki, M., & Well, R. (2020). N_2_O isotope approaches for source partitioning of N_2_O production and estimation of N_2_O reduction-validation with the ^15^N gas-flux method in laboratory and field studies. *Biogeosciences*, *17*(22), 5513–5537. https://doi.org/10.5194/bg-17-5513-2020

Lewicka-Szczebak, D., Well, R., Köster, J. R., Fuß, R., Senbayram, M., Dittert, K., & Flessa, H. (2014). Experimental determinations of isotopic fractionation factors associated with N_2_O production and reduction during denitrification in soils. *Geochimica et Cosmochimica Acta*, *134*, 55–73. <https://doi.org/10.1016/j.gca.2014.03.010>

Lewicka-Szczebak, D., Lewicki, M. P., Well, R. (2020). N_2_O isotope approaches for source partitioning of N_2_O production and estimation of N_2_O reduction – validation with the ^15^N gas-flux method in laboratory and field studies. *Biogeosciences*, 17, 5513–5537. https://doi.org/10.5194/bg-17-5513-2020

Mo, D., Liu, Y., Li, M., Li, H., Zhang, J., Qian, H., Ding, Y., & Jiang, Y. (2025). Warming increases CH_4_ emissions from rice paddies through shifts in methanogenic and methanotrophic communities. *Soil Biology and Biochemistry*, *200*(August 2024), 109639. <https://doi.org/10.1016/j.soilbio.2024.109639>

Mitsuta, A., Lourenço, K. S., de Oliveira, B., G., de Assis Costa, O. Y., Cantarella, H., Kuramae, E. E. (2025). Soil pH determines the shift of key microbial energy metabolic pathways associated with soil nutrient cycle. Applied Soil Ecology, 208, 105992. https://doi.org/10.1016/j.apsoil.2025.105992

Mushinski, R. M., Phillips, R. P., Payne, Z. C., Abney, R. B., Jo, I., Fei, S., Pusede, S. E., White, J. R., Rusch, D. B., & Raff, J. D. (2019). Microbial mechanisms and ecosystem flux estimation for aerobic NO y emissions from deciduous forest soils. *Proceedings of the National Academy of Sciences of the United States of America*, *116*(6), 2138–2145. https://doi.org/10.1073/pnas.1814632116

Nicol, G. W., Leininger, S., Schleper, C., & Prosser, J. I. (2008). The influence of soil pH on the diversity, abundance and transcriptional activity of ammonia oxidizing archaea and bacteria. *Environmental Microbiology*, *10*(11), 2966–2978. https://doi.org/10.1111/j.1462-2920.2008.01701.x

Norton, J. M., & Stark, J. M. (2011). Regulation and measurement of nitrification in terrestrial systems. *Methods in Enzymology*, *486*(C), 343–368. https://doi.org/10.1016/B978-0-12-381294-0.00015-8

Rotthauwe, J. H., Witzel, K. P., & Liesack, W. (1997). The ammonia monooxygenase structural gene amoa as a functional marker: Molecular fine-scale analysis of natural ammonia-oxidizing populations. *Applied and Environmental Microbiology*, *63*(12), 4704–4712. https://doi.org/10.1128/aem.63.12.4704-4712.1997

Scala, D. J., & Kerkhof, L. J. (1998). Nitrous oxide reductase (nosZ) gene-specific PCR primers for detection of denitrifiers and three nosZ genes from marine sediments. *FEMS Microbiology Letters*, *162*(1), 61–68. https://doi.org/10.1016/S0378-1097(98)00103-7

Steinberg, L. M., & Regan, J. M. (2009). mcrA-targeted real-time quantitative PCR method to examine methanogen communities. *Applied and Environmental Microbiology*, *75*(13), 4435–4442. https://doi.org/10.1128/AEM.02858-08

Teng, J., Hou, R., Dungait, J. A. J., Zhou, G., Kuzyakov, Y., Zhang, J., Tian, J., Cui, Z., Zhang, F., & Delgado-Baquerizo, M. (2024). Conservation agriculture improves soil health and sustains crop yields after long-term warming. *Nature Communications*, *15*(1), 8785. https://doi.org/10.1038/s41467-024-53169-6

Throbäck, I. N., Enwall, K., Jarvis, Å., & Hallin, S. (2004). Reassessing PCR primers targeting nirS, nirK and nosZ genes for community surveys of denitrifying bacteria with DGGE. *FEMS Microbiology Ecology*, *49*(3), 401–417. https://doi.org/10.1016/j.femsec.2004.04.011

Toyoda, S., Mutobe, H., Yamagishi, H., Yoshida, N., & Tanji, Y. (2005). Fractionation of N2O isotopomers during production by denitrifier. *Soil Biology and Biochemistry*, *37*(8), 1535–1545. https://doi.org/10.1016/j.soilbio.2005.01.009

Wu, D., Well, R., Cárdenas, L. M., Fuß, R., Lewicka-Szczebak, D., Köster, J. R., Brüggemann, N., & Bol, R. (2019). Quantifying N_2_O reduction to N_2_ during denitrification in soils via isotopic mapping approach: Model evaluation and uncertainty analysis. *Environmental Research*, *179*(October). https://doi.org/10.1016/j.envres.2019.108806

Yang, X., Xiong, J., Du, T., Ju, X., Gan, Y., Li, S., Xia, L., Shen, Y., Pacenka, S., Steenhuis, T. S., Siddique, K. H. M., Kang, S., & Butterbach-Bahl, K. (2024). Diversifying crop rotation increases food production, reduces net greenhouse gas emissions and improves soil health. *Nature Communications*, *15*(1). <https://doi.org/10.1038/s41467-023-44464-9>

Yu, L., Harris, E., Lewicka-Szczebak, D., Barthel, M., Blomberg, M. R. A., Harris, S. J., Johnson, M. S., Lehmann, M. F., Liisberg, J., Müller, C., Ostrom, N. E., Six, J., Toyoda, S., Yoshida, N., & Mohn, J. (2020). What can we learn from N_2_O isotope data? Analytics, processes and modelling. *Rapid Communications in Mass Spectrometry*, *34*(20), e8858. https://doi.org/10.1002/rcm.8858

Zou, J., Huang, Y., Jiang, J., Zheng, X., & Sass, R. L. (2005). A 3-year field measurement of methane and nitrous oxide emissions from rice paddies in China: Effects of water regime, crop residue, and fertilizer application. *Global Biogeochemical Cycles*, *19*(2), 1–9. https://doi.org/10.1029/2004GB002401
